# Effects of traditional Chinese exercise on lung function and mental health in patients with COPD: a systematic review and meta-analysis

**DOI:** 10.3389/fpubh.2025.1612741

**Published:** 2025-07-10

**Authors:** Shuning Liu, Debin Xu

**Affiliations:** School of Marxism, Changchun University of Chinese Medicine, Changchun, China

**Keywords:** COPD, traditional Chinese exercise, lung function, mental health, systematic review

## Abstract

**Objective:**

Despite their widespread use in the management of chronic obstructive pulmonary disease (COPD), pharmacological treatments often demonstrate limited efficacy in alleviating symptoms such as dyspnea and psychological pressure. These limitations highlight the need for complementary nonpharmacological interventions. This study aimed to evaluate the efficacy of traditional Chinese exercise (TCE) in improving lung function and mental health among patients with COPD.

**Methods:**

We conducted a comprehensive search across several databases: CNKI (1979–2024), Wanfang (1998–2024), PubMed (1966–2024), the Cochrane Library (1999–2024), and Web of Science (1961–2024), up to September 2024, to gather all randomized controlled trials (RCTs) studies that evaluated the effects of TCE as the primary intervention for patients with COPD. The results were analyzed and presented using Review Manager 5.4 software, ensuring a systematic approach to data interpretation and visualization.

**Results:**

67 studies were included and encompassing 5,475 patients. The meta-analysis demonstrated that TCE significantly improved various outcomes in COPD patients, including FEV1 [MD = 0.28, 95% CI (0.23, 0.33), *p* < 0.001], FEV1% [MD = 5.53, 95% CI (4.41, 6.65), *p* < 0.001], FVC [MD = 0.31, 95% CI (0.29, 0.34), *p* < 0.001], FEV1/FVC (%) [MD = 6.00, 95% CI (4.27, 7.73), *p* < 0.001], the 6MWT [MD = 42.14, 95% CI (36.54, 47.73), *p* < 0.001], CAT [MD = −4.20, 95% CI (−4.74, −3.66), *p* < 0.001], anxiety [MD = −1.26, 95% CI (−1.64, −0.89), *p* < 0.001], and depression [MD = −1.26, 95% CI (−1.59, −0.94), *p* < 0.001].

**Conclusion:**

TCE significantly improved lung function and alleviated anxiety and depression in COPD patients. This study not only highlights the value of TCE as a nonpharmacological intervention but also offers new directions for psychological management, warranting broader implementation.

**Systematic review registration:**

CRD42024586079, https://www.crd.york.ac.uk/PROSPERO/view/CRD42024586079.

## 1 Introduction

Chronic obstructive pulmonary disease (COPD) is a significant global public health concern that profoundly affects patients’ quality of life, and the incidence rate among individuals over 40 years old globally has reached 10% (1). The progressive and irreversible nature of persistent airflow limitation makes daily activities increasingly challenging. Additionally, the prevalence of COPD among individuals aged 40 and older has reached 10%, making it the fourth leading cause of death from respiratory diseases (2). COPD presents with sudden onset, persistent, and recurrent symptoms, which not only impose psychological burdens but also exacerbate anxiety and depression, leading to a decrease in confidence in recovery. Furthermore, the substantial medical resources required to manage COPD contribute to an increased socioeconomic burden. Currently, standardized rehabilitation for COPD is still being explored, with pulmonary rehabilitation considered a crucial part of comprehensive care, with a primary focus on exercise and endurance training (3). However, COPD patients frequently experience shortness of breath, weakness, and limited physical activity, which can entrap them in a vicious cycle of inactivity. Therefore, finding an efficient exercise program with moderate to low intensity has become a pressing need.

Traditional Chinese exercise (TCE), which includes Baduanjin, Taichi, Liuzijue, Wuqinxi, and Yijinjing, is characterized by slow, smooth, gentle movements that have a low impact (4). These exercises combine breath control, psychological awareness, mental focus, and physical activity to promote greater mental calmness and a sense of inner well-being. TCEs typically require no specialized equipment and can be practiced in small indoor spaces or outdoor settings such as parks, making them highly accessible. A typical exercise lasts 20 to 40 min, with a recommended frequency of 3 to 5 times per week, and different training plans can be developed according to the patient’s needs. While some forms such as Baduanjin and Liuzijue are simple enough to be self-taught using instructional videos or classes, others like Taichi, Wuqinxi and Yijinjing may require initial coaching from trained instructors to ensure proper technique. As a multi-modal mind–body intervention, TCE aligns well with the exercise needs of COPD patients. Research has shown that TCE can serve as an alternative therapy to improve lung function, flexibility and balance, quality of life, and mental health in COPD patients (5). However, individual studies are often affected by differences in sample size, inclusion criteria, and research methods, resulting in a lack of robust evidence-based research on the efficacy of different types of TCE for treating COPD. This variability makes it challenging to guide clinical practice effectively.

In this study, we implemented an extensive meta-analysis to investigate the impact of TCE as an alternative treatment on the lung function and mental health of patients with COPD. This research fills a gap in the literature and provides new insights and robust psychological intervention strategies for the supportive treatment of COPD.

## 2 Methods

### 2.1 Registration

This study was conducted in accordance with the PRISMA statement and has been registered with PROSPERO (CRD42024586079). The PICO tool was used to develop the search strategy, where the *Population* of interest was patients with COPD, the *Intervention* was TCEs, the *Comparison* was usual care or daily activities, and the *Outcomes* were lung function, health status, and mental health.

### 2.2 Search strategy

Two researchers (S. N. Liu and D. B. Xu) independently searched five databases, including CNKI (1979–2024), Wanfang (1998–2024), PubMed (1966–2024), the Cochrane Library (1999–2024), and Web of Science (1961–2024), for randomized controlled trial (RCT) exploring the effects of TCE on patients with COPD up to September 2024. Since all reviewed studies were published, there was no requirement for ethical approval or patient consent. The search strategy was created by a combination of medical subject heading (MeSH) terms and free words, in which the MeSH terms are “Pulmonary Disease, Chronic Obstructive OR COPD” and “Taichi,” “Qigong.” Free words are synonyms of each subject word, including “Traditional Chinese Exercise,” “Baduanjin,” “Taiji,” “Liuzijue,” “Wuqinxi,” and “Yijinjing.” All databases were searched in any language. In cases of disagreement between the two researchers, a third researcher was consulted to reach a resolution.

### 2.3 Inclusion and exclusion criteria

The inclusion criteria included: (1) the study type was an RCT assessing the effects of various TCE on COPD; (2) the experimental group employed TCE (Baduanjin, Taichi, Liuzijue, Wuqinxi, or Yijinjing), whereas the control group received standard treatment, with or without additional exercise interventions; (3) participants were COPD patients diagnosed according to the COPD guidelines of the Chinese Medical Association Respiratory Diseases Society or Global Initiative for Chronic Obstructive Lung Disease (6); (4) multiple outcomes or indicators were used to assess the efficacy of TCE; (5) the experimental outcomes or indicators included one of the following measures: lung function (FEV1, FEV1%, FEV1/FVC%, FVC), health status (6MWT, CAT), and mental health (SAS, SDS, HAM-A, HAM-D, HADS).

The exclusion criteria included: (1) reviews, letters, conference abstracts, and similar types of publications; (2) non-RCT designs, case reports, dissertations, or animal studies; (3) studies with incomplete data that could not be extracted for the calculation of mean values and standard deviations; and (4) studies with patients lacking general exercise capacity or compliance, making it difficult to cooperate with training, evaluation, and treatment.

### 2.4 Study selection and data extraction

Two researchers (S. N. Liu and D. B. Xu) independently examined all the studies. After removing duplicates according to predefined criteria, they independently screened and excluded studies that did not meet the inclusion criteria. The following information was extracted from the remaining studies: authors, publication year, demographic characteristics, specific intervention and control plans, measurement methods, and outcome indicators. In cases of disagreement between the two researchers, a third researcher was consulted to reach a resolution.

### 2.5 Quality appraisal

Two researchers (S. N. Liu and D. B. Xu) independently assessed the risk of bias for all the retrieved studies using the Cochrane Collaboration tool, and if the results were inconsistent, a third researcher was consulted to reach a resolution. The quality assessment of the included studies was represented with “low risk of bias” (+) displayed in green, “high risk of bias” (−) displayed in red, and “unclear risk of bias” (?) displayed in yellow (7).

### 2.6 Data synthesis and analysis

The analysis and exhibition of the survey results in this study were executed using Manager 5.4 software. The lung function and mental health outcomes examined are continuous variables, analyzed using mean difference (MD) and standardized mean difference (SMD), each accompanied by a 95% confidence interval (CI). To assess statistical heterogeneity, Chi-square tests and *I*^2^ statistics were applied. A fixed-effects model was selected when heterogeneity is not significant (*I*^2^ < 50% and/or *p* > 0.05). Conversely, when heterogeneity is significant (*I*^2^ ≥ 50% and/or *p* ≤ 0.05), a random-effects model was employed, along with subgroup analyses on the basis of exercise duration and frequency, to identify the sources of heterogeneity (8). Furthermore, *p* ≤ 0.05 indicates a significant difference, demonstrating statistical significance in the meta-analysis results.

## 3 Results

### 3.1 Study characteristics

**Figure 1:**
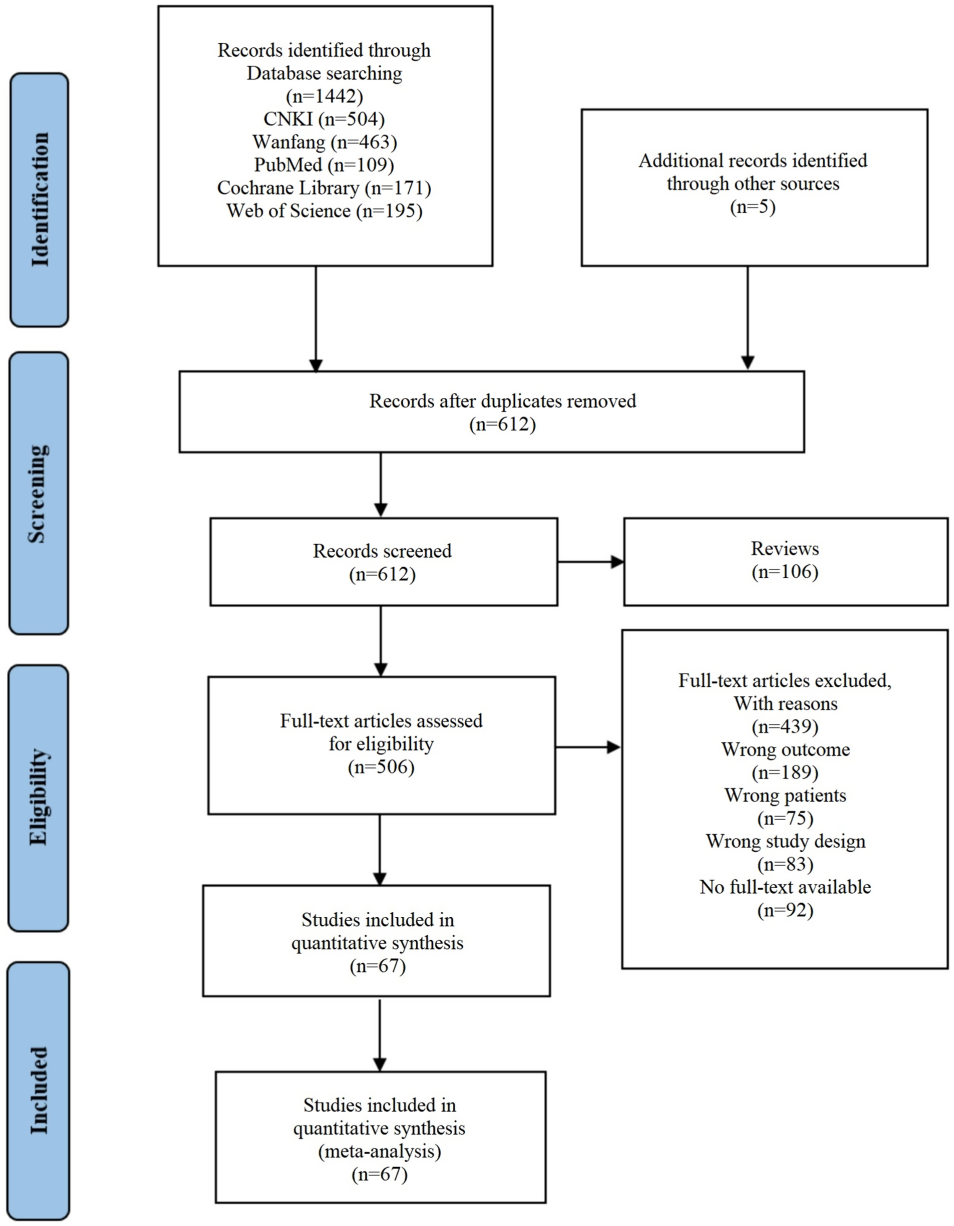
Flow diagram of the literature screening process.

This study included 67 studies published in China between 2008 and 2024 (Figure 1). A total of 5,475 participants were involved, with 2,745 in the treatment group and 2,730 in the control group; participants’ ages ranged from 45 to 82 years, with intervention durations ranging from 8 to 108 weeks, frequencies ranging from 1 to 7 times per week, and durations ranging from 15 to 90 min per session. Among the 67 eligible studies, 20 focused on Baduanjin, 18 on Taichi, 18 on Liuzijue, 9 on Wuqinxi, and 2 on Yijinjing. Among them, 19 studies utilized follow-up assessments to determine the benefits of TCE on anxiety and depression. Supplementary Table S1 provides a comprehensive overview of these RCTs.

### 3.2 Risk of bias assessment

**Figure 2:**
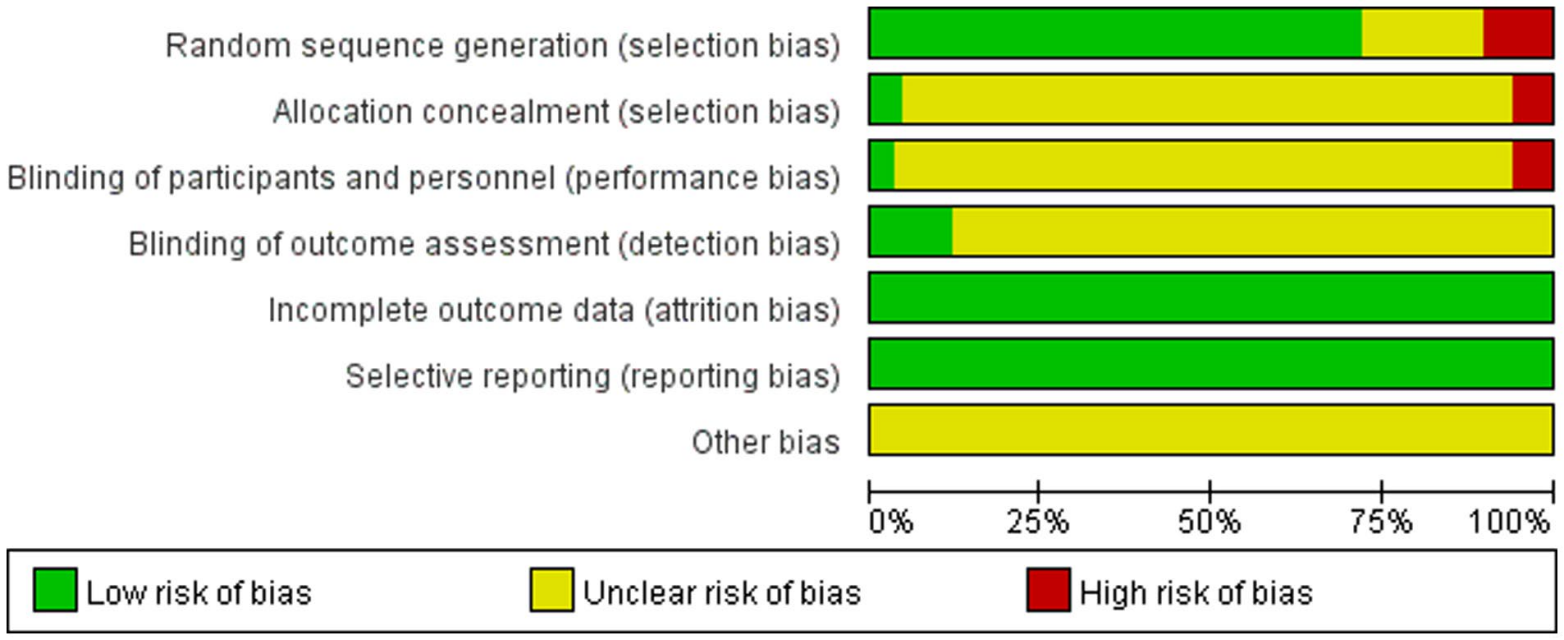
Risk of bias assessment.

**Figure 3:**
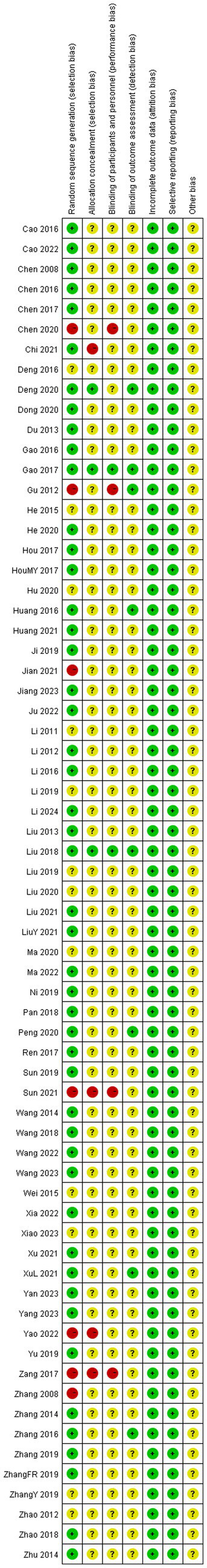
Summary of bias risk for each bias risk.

The assessment of bias risk reached a consensus after discussion (9–75). For random sequence generation, 48 studies utilized randomization methods and were evaluated as having a low risk of bias. Of the remaining 19 studies, 12 did not mention the randomization method (17, 31, 35, 36, 39, 41, 46, 63, 66, 68, 71, 72), and 7 grouped patients by age or sex, resulting in a high-risk rating (24, 26, 29, 33, 53, 70, 73). With respect to allocation concealment, 3 studies provided detailed descriptions and were rated as low risk (50, 56, 67), whereas 4 were open-label trials and received a high-risk rating (24, 30, 70, 73). Four studies did not employ blinding and were considered high risk (29, 33, 70, 73), whereas 8 studies applied blinding during outcome assessment and were deemed to have a low risk of bias (13, 33, 43, 50, 56, 61, 67, 75). Outcome data and selective reporting were complete across all studies, indicating low risk. For the category of “other biases,” none of the studies provided details, and they were rated as having an unclear risk (Figures 2, 3).

### 3.3 Results of the meta-analysis

#### 3.3.1 FEV1 (L)

Among the 45 included studies (9, 11, 13, 14, 16, 18–23, 25, 28, 30, 33–35, 37–40, 42–47, 49–51, 55–59, 61–64, 67, 68, 70, 72, 74, 75), the effects of TCE on FEV1 in patients with COPD were reported. These studies examined five different types of exercises: Baduanjin, Taichi, Liuzijue, Wuqinxi, and Yijinjing. The experimental group consisted of 1,895 participants, while the control group included 1,883 participants. The random-effects model indicated no statistical heterogeneity in the Liuzijue and Yijinjing groups (*p* = 0.45, *I*^2^ = 0%), (*p* = 0.76, *I*^2^ = 0%). All TCE groups showed significantly higher FEV1 compared to the control group. Results for each group included Baduanjin [MD = 0.32, 95% CI (0.25, 0.40), *p* < 0.001], Taichi [MD = 0.21, 95% CI (0.12, 0.30), *p* < 0.001], Liuzijue [MD = 0.21, 95% CI (0.16, 0.25), *p* < 0.001], Wuqinxi [MD = 0.42, 95% CI (−0.11, 0.73), *p* < 0.001], and Yijinjing [MD = 0.54, 95% CI (0.36, 0.73), *p* < 0.001].

**Figure 4:**
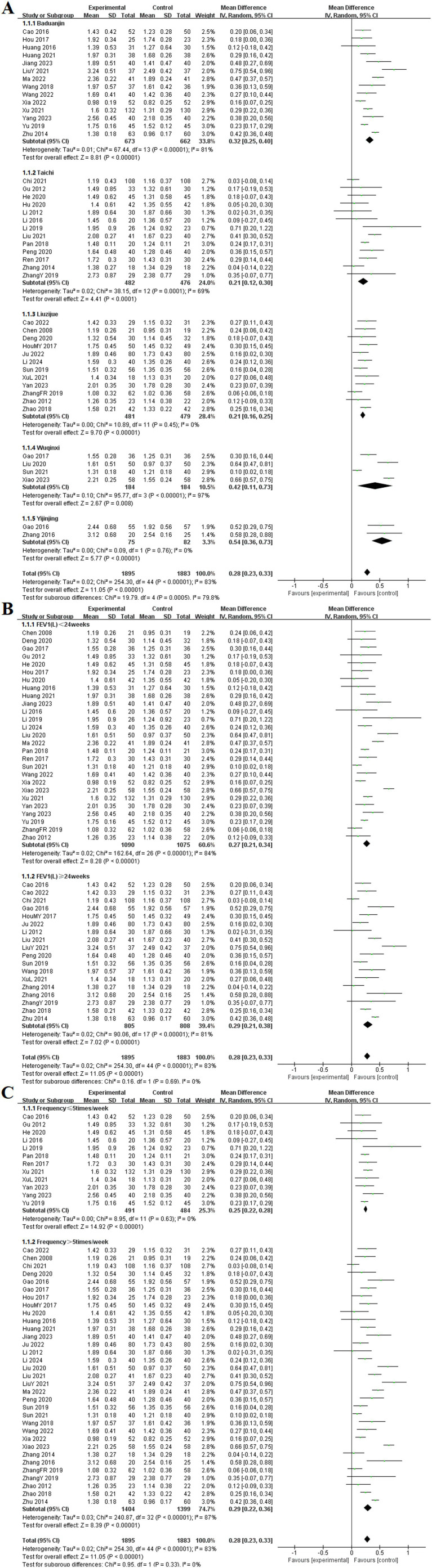
Effect of TCE on FEV1 in patients with COPD. **(A)** Types of intervention; **(B)** Duration of intervention; **(C)** Frequency of intervention.

Subgroup analyses were conducted based on intervention duration and frequency. In the analysis of intervention duration, the experimental group exhibited a significantly higher FEV1than did the control group before 24 weeks of TCE [MD = 0.27, 95% CI (0.21, 0.34), *p* < 0.001]. Similarly, for interventions lasting 24 weeks or longer, the experimental group demonstrated a statistically significant improvement in FEV1 [MD = 0.29, 95% CI (0.21, 0.38), *p* < 0.001]. Additionally, no significant heterogeneity was observed in the subgroup with a frequency of ≤ 5 times per week (*p* = 0.63, *I*^2^ = 0%). Significant differences were found at frequencies of ≤ 5 times per week [MD = 0.25, 95% CI (0.22, 0.28), *p* < 0.001] and > 5 times per week [MD = 0.29, 95% CI (0.26, 0.32), *p* < 0.001] (Figure 4).

#### 3.3.2 FEV1 (%)

Among the 35 included studies (9, 12, 15, 17, 19, 23, 25, 27–29, 31–33, 35, 38, 41, 42, 45–50, 52, 53, 59–61, 63, 67, 68, 71, 73–75), the effects of TCE on FEV1% in patients with COPD were reported. These studies focused on five specific exercise forms: Baduanjin, Taichi, Liuzijue, Wuqinxi, and Yijinjing. The experimental group consisted of 1,270 participants, while the control group included 1,274 participants. The random-effects model indicated no statistical heterogeneity in the Taichi and Liuzijue groups (*p* = 0.06, *I*^2^ = 44%), (*p* = 0.32, *I*^2^ = 13%). However, except for the Yijinjing group, all TCE groups showed significantly higher FEV1% compared to the control group. The results for each group included Baduanjin [MD = 6.26, 95% CI (3.71, 8.81), *p* < 0.001], Taichi [MD = 3.81, 95% CI (2.59, 5.02), *p* < 0.001], Liuzijue [MD = 8.19, 95% CI (6.39, 9.99), *p* < 0.001], Wuqinxi [MD = 4.70, 95% CI (0.74, 8.67), *p* < 0.002], and Yijinjing [MD = 7.30, 95% CI (−0.60, 15.20), *p* = 0.07].

**Figure 5:**
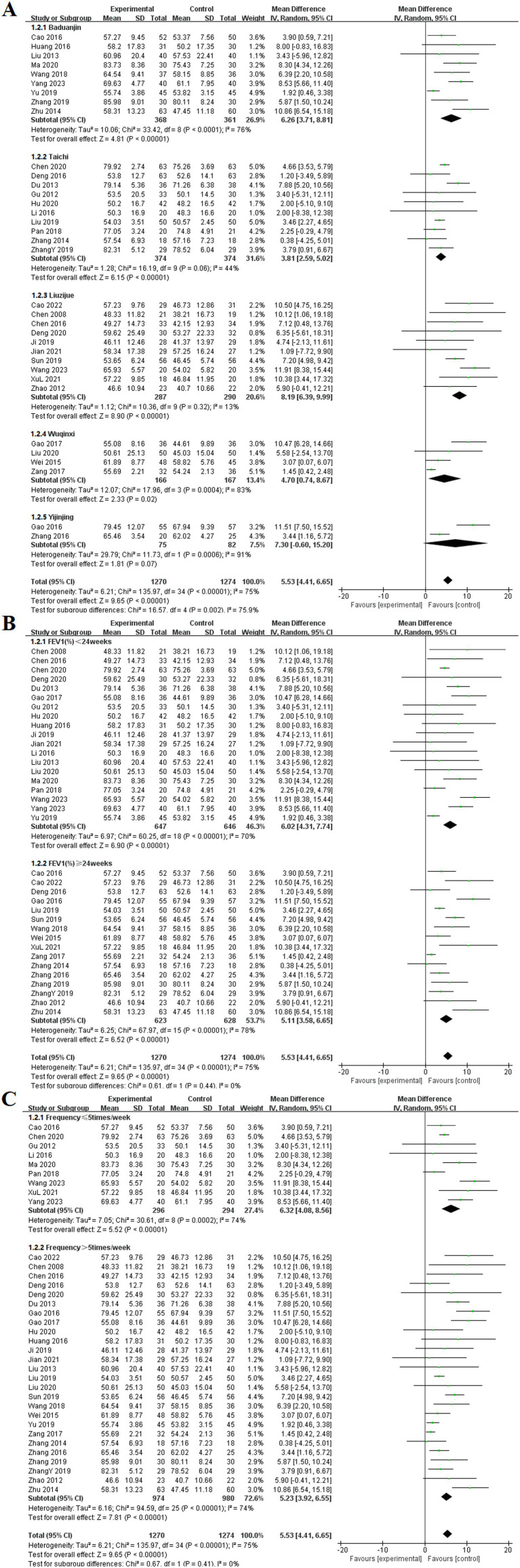
Effect of TCE on FEV1% in patients with COPD. **(A)** Types of intervention; **(B)** Duration of intervention; **(C)** Frequency of intervention.

Subgroup analyses were performed based on both intervention duration and frequency. In the analysis based on intervention duration, the experimental group exhibited a significantly higher FEV1% than did the control group before 24 weeks of TCE [MD = 6.02, 95% CI (4.31, 7.74), *p* < 0.001]. Similarly, for interventions lasting 24 weeks or longer, the experimental group demonstrated a statistically significant improvement in FEV1% [MD = 5.11, 95% CI (3.58, 6.65), *p* < 0.001]. The subgroup analysis based on exercise frequency indicated that both groups in the experimental cohort, significant differences at frequencies of ≤ 5 times per week [MD = 6.32, 95% CI (4.08, 8.56), *p* < 0.001] and > 5 times per week [MD = 5.23, 95% CI (3.92, 6.55), *p* < 0.001] (Figure 5).

#### 3.3.3 FVC

**Figure 6:**
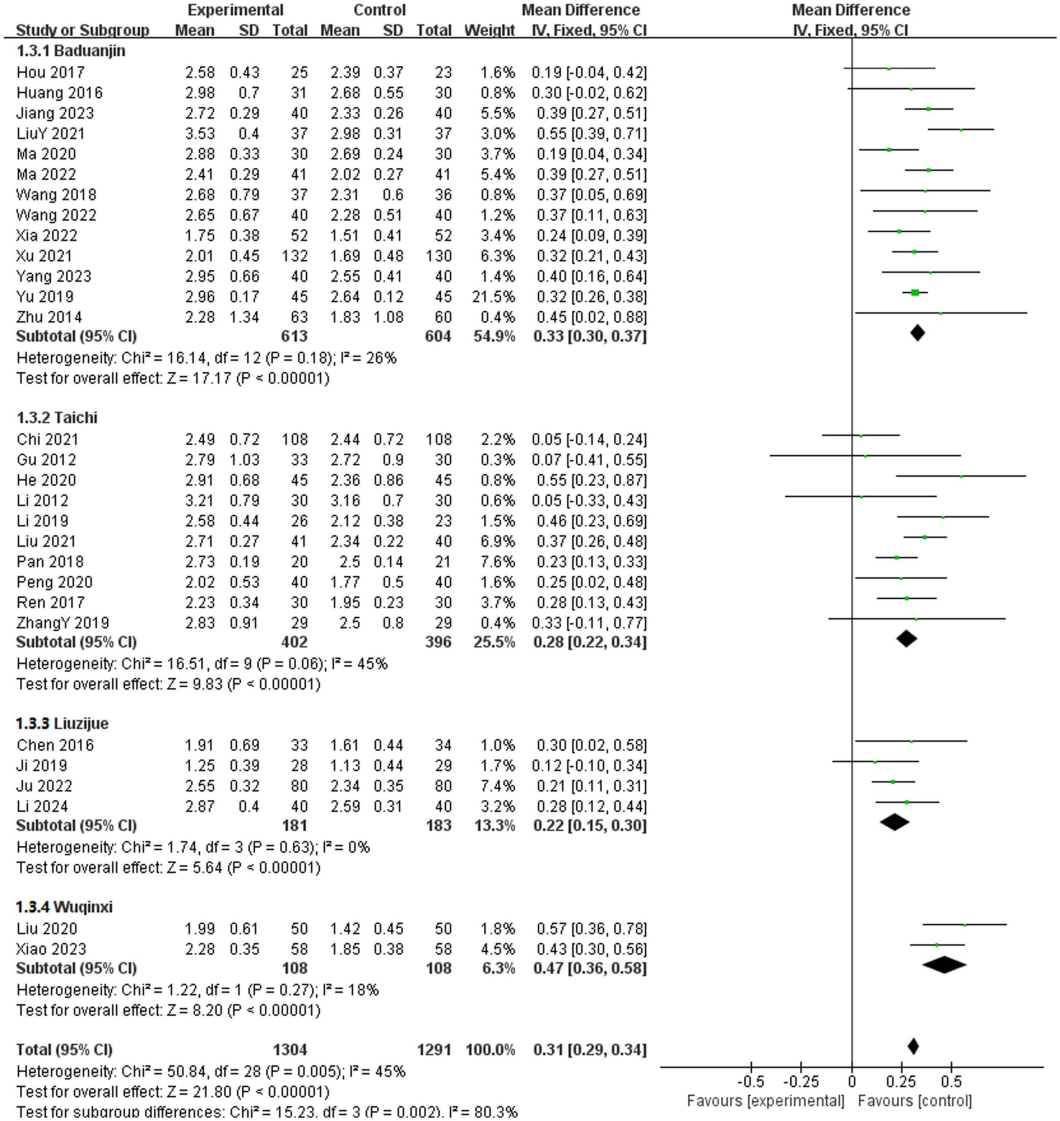
Effect of TCE on FVC in patients with COPD. Types of intervention.

In the included literature, 29 studies reported the effects of TCE on FVC in patients with COPD (11, 12, 14, 16–23, 25, 28, 30, 33, 34, 37, 39, 40, 42–44, 48, 52, 57, 58, 67, 68, 72). These studies encompassed four exercise methods: Baduanjin, Taichi, Liuzijue, and Wuqinxi. The experimental group comprised 1,304 participants, whereas the control group included 1,291 participants. The fixed-effects model indicated no statistical heterogeneity in the Baduanjin, Taichi, Liuzijue, and Wuqinxi groups (*p* = 0.18, *I*^2^ = 26%), (*p* = 0.06, *I*^2^ = 45%), (*p* = 0.63, *I*^2^ = 0%), (*p* = 0.27, *I*^2^ = 18%). Compared with the control group, all TCE groups presented a significantly greater FVC. The results for each group included Baduanjin [MD = 0.33, 95% CI (0.30, 0.37), *p* < 0.001], Taichi [MD = 0.28, 95% CI (0.22, 0.34), *p* < 0.001], Liuzijue [MD = 0.22, 95% CI (0.15, 0.30), *p* < 0.001], and Wuqinxi [MD = 0.47, 95% CI (0.36, 0.58), *p* < 0.001] (Figure 6).

#### 3.3.4 FEV1/FVC (%)

Among the 43 included studies (9, 11, 12, 14, 15, 17, 19, 21, 23, 27, 28, 30, 32–35, 37–41, 43–45, 47–52, 55–57, 61, 62, 66–68, 70–72, 74, 75), the effects of TCE on FEV1/FVC (%) in patients with COPD were reported. These studies encompassed five types of exercises: Baduanjin, Taichi, Liuzijue, Wuqinxi, and Yijinjing. The experimental group consisted of 1,793 participants, whereas the control group included 1,783 participants. The random-effects model indicated that all TCE groups showed significantly higher FEV1/FVC (%) compared to the control group. Results for each group included Baduanjin [MD = 6.98, 95% CI (4.88, 9.07), *p* < 0.001], Taichi [MD = 3.89, 95% CI (1.36, 6.42), *p* < 0.001], Liuzijue [MD = 5.91, 95% CI (3.66, 8.15), *p* < 0.001], Wuqinxi [MD = 9.54, 95% CI (3.13, 15.95), *p* < 0.004], and Yijinjing [MD = 4.46, 95% CI (2.55, 6.36), *p* < 0.001].

**Figure 7:**
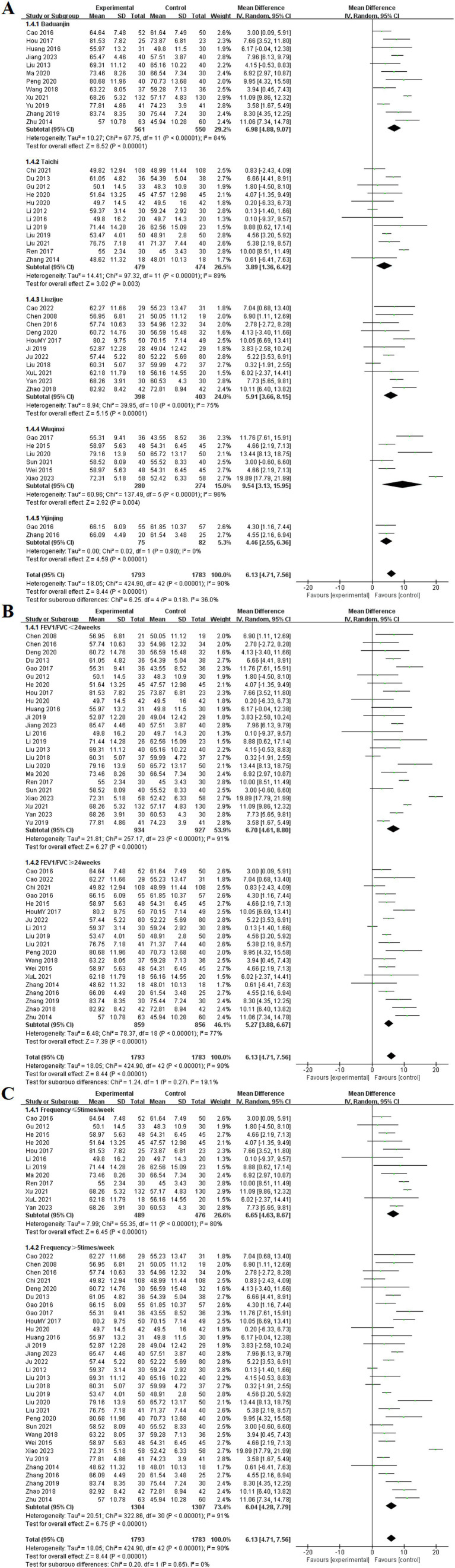
Effect of TCE on FEV1/FVC (%) in patients with COPD. **(A)** Types of intervention; **(B)** Duration of intervention; **(C)** Frequency of intervention.

Subgroup analyses were conducted on the basis of intervention duration and frequency. In the analysis of intervention duration, the experimental group exhibited a significantly higher FEV1/FVC (%) than did the control group before 24 weeks of TCE [MD = 6.70, 95% CI (4.61, 8.80), *p* < 0.001]. Similarly, for interventions lasting 24 weeks or longer, the experimental group demonstrated a statistically significant improvement in FEV1/FVC (%) [MD = 5.27, 95% CI (3.88, 6.67), *p* < 0.001]. The subgroup analysis based on exercise frequency indicated that both groups in the experimental cohort, those exercising ≤ 5 times per week and those exercising > 5 times per week, achieved significantly higher FEV1/FVC (%) than the control group, with statistically significant differences of [MD = 6.65, 95% CI (4.63, 8.67), *p* < 0.001] and [MD = 6.04, 95% CI (4.28, 7.79), *p* < 0.001], respectively (Figure 7).

#### 3.3.5 6MWT

Among the included studies, 40 articles reported the effects of TCE on the 6MWT in patients with COPD (11, 13–15, 17–21, 23–25, 27–29, 31–34, 38, 40–45, 57, 59–65, 67, 68, 72–75). These studies investigated five types of exercises: Baduanjin, Taichi, Liuzijue, Wuqinxi, and Yijinjing. The experimental group consisted of 1,701 participants, whereas the control group included 1,695 participants. The random-effects model showed no statistical heterogeneity among the Liuzijue, Wuqinxi, and Yijinjing groups (*p* = 0.28, *I*^2^ = 20%; *p* = 0.14, *I*^2^ = 43%), (*p* = 0.41, *I*^2^ = 0%). Each TCE group demonstrated significantly higher 6MWT results compared to the control group, specifically Baduanjin [MD = 45.35, 95% CI (37.39, 53.30), *p* < 0.001], Taichi [MD = 42.57, 95% CI (31.51, 53.64), *p* < 0.001], Liuzijue [MD = 39.18, 95% CI (30.75, 47.62), *p* < 0.001], Wuqinxi [MD = 45.72, 95% CI (38.34, 53.10), *p* < 0.004], and Yijinjing [MD = 15.98, 95% CI (11.72, 20.23), *p* < 0.001].

**Figure 8:**
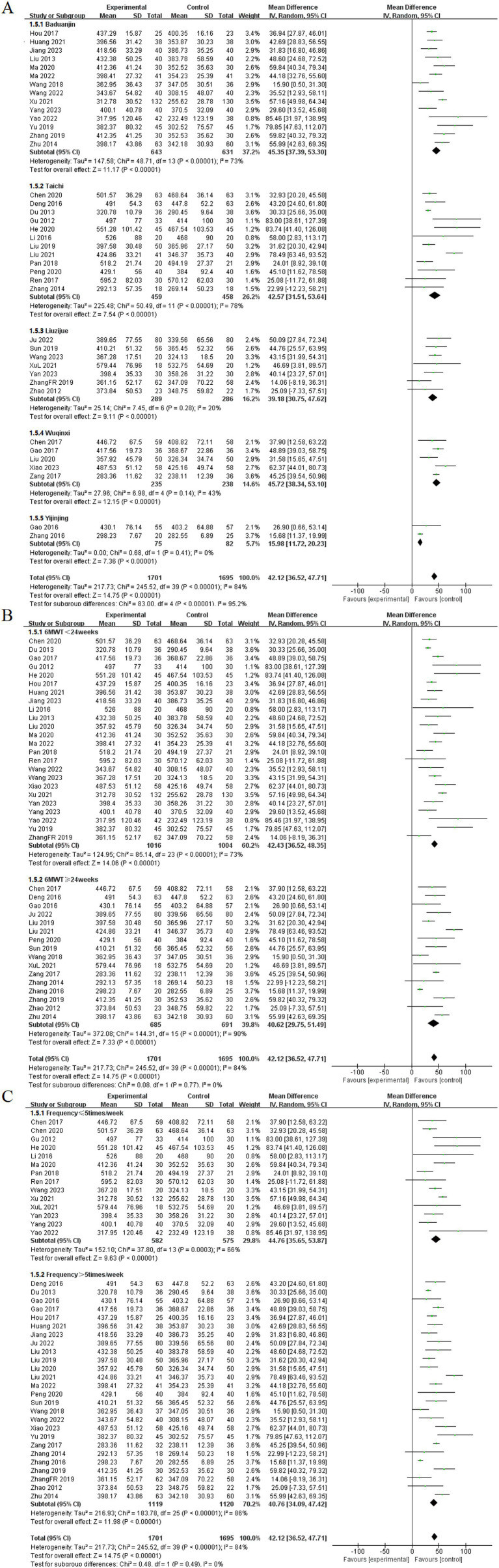
Effect of TCE on 6MWT in patients with COPD. **(A)** Types of intervention; **(B)** Duration of intervention; **(C)** Frequency of intervention.

Subgroup analyses were conducted on the basis of intervention duration and frequency. In the analysis of intervention duration, the experimental group exhibited a significantly higher 6MWT than did the control group before 24 weeks of TCE [MD = 42.43, 95% CI (36.52, 48.35), *p* < 0.001]. Similarly, for interventions lasting 24 weeks or longer, the experimental group demonstrated a statistically significant improvement in 6MWT [MD = 40.62, 95% CI (36.52, 47.71), *p* < 0.001]. The subgroup analysis based on exercise frequency indicated that both groups in the experimental cohort presented statistical differences at frequencies of ≤ 5 times per week [MD = 44.76, 95% CI (35.65, 53.87), *p* < 0.001] and > 5 times per week [MD = 40.76, 95% CI (34.09, 47.42), p < 0.001] (Figure 8).

#### 3.3.6 CAT

Among the included studies, 21 articles reported the effects of TCE on the CAT in patients with COPD (10, 13, 14, 16, 24, 25, 29, 32, 41–45, 48, 56, 57, 60, 61, 66, 70, 73). These studies encompassed four types of exercises: Baduanjin, Taichi, Liuzijue, and Wuqinxi. The experimental group consisted of 802 participants, whereas the control group included 805 participants. The random-effects model showed no statistical heterogeneity among the Taichi and Wuqinxi groups (*p* = 0.13, *I*^2^ = 39%), (*p* = 0.33, *I*^2^ = 11%). Each TCE groups showed significantly lower CAT compared to the control group. The results for each group included Baduanjin [MD = −4.79, 95% CI (−5.68, −3.90), *p* < 0.001], Taichi [MD = −3.86, 95% CI (−4.48, −3.23), *p* < 0.001], Liuzijue [MD = −3.76, 95% CI (−5.01, −2.51), *p* < 0.001], and Wuqinxi [MD = −3.94, 95% CI (−5.38, −2.50), *p* < 0.001].

**Figure 9:**
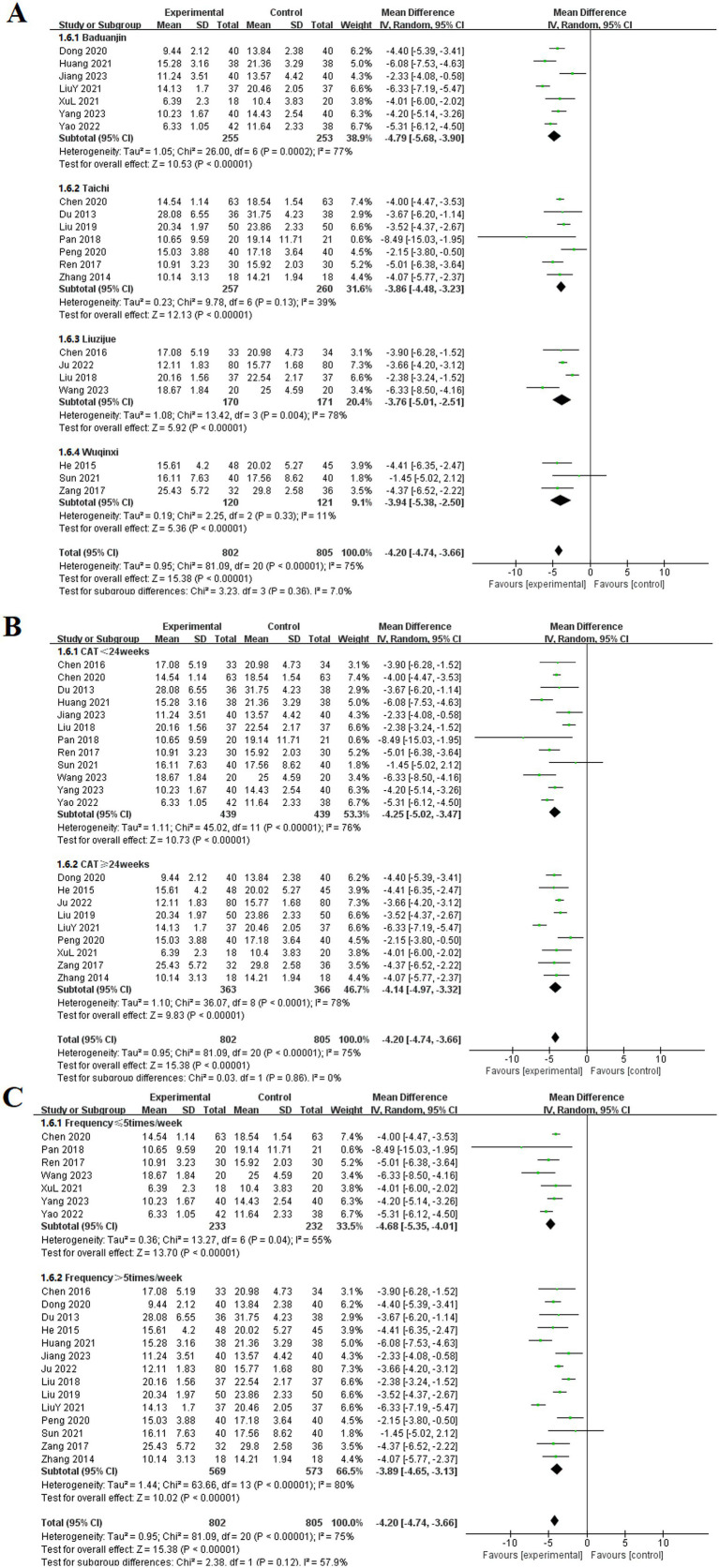
Effect of TCE on CAT in patients with COPD. **(A)** Types of intervention; **(B)** Duration of intervention; **(C)** Frequency of intervention.

Subgroup analyses were performed on the basis of intervention duration and frequency. In the subgroup analysis based on intervention duration, the experimental group exhibited a significantly lower CAT than did the control group before 24 weeks of TCE [MD = −4.25, 95% CI (−5.02, −3.47), *p* < 0.001]. Similarly, for interventions lasting 24 weeks or longer, the experimental group demonstrated a statistically significant improvement in CAT [MD = −4.14, 95% CI (−4.97, −3.32), *p* < 0.001]. The subgroup analysis based on exercise frequency indicated that both groups in the experimental cohort, those exercising ≤ 5 times per week and those exercising > 5 times per week, achieved significantly lower CAT than did the control group, with statistically significant differences of [MD = −4.68, 95% CI (−5.35, −4.01), *p* < 0.001] and [MD = −3.89, 95% CI (−4.65, −3.13), *p* < 0.001], respectively (Figure 9).

#### 3.3.7 Anxiety

Among the 19 included studies (9, 10, 14, 21, 22, 24–26, 36, 42, 44, 48, 53–56, 65, 66, 69), the effects of TCE on anxiety in patients with COPD were reported. These studies involved four types of exercises: Baduanjin, Taichi, Liuzijue, and Wuqinxi. The experimental group consisted of 816 participants, whereas the control group included 807 participants. The studies utilized SMD to compile data on anxiety symptoms using assessment tools such as the SAS, HAM-A, and HADS. The random-effects model indicated that all TCE groups showed significantly lower anxiety than did the control group. The results for each group included Baduanjin [MD = −1.62, 95% CI (−2.31, −0.94), *p* < 0.001], Taichi [MD = −0.54, 95% CI (−1.28, 0.20), p < 0.001], Liuzijue [MD = −1.03, 95% CI (−1.46, −0.59), *p* < 0.001], and Wuqinxi [MD = −1.44, 95% CI (−2.35, −0.52), *p* < 0.001].

**Figure 10:**
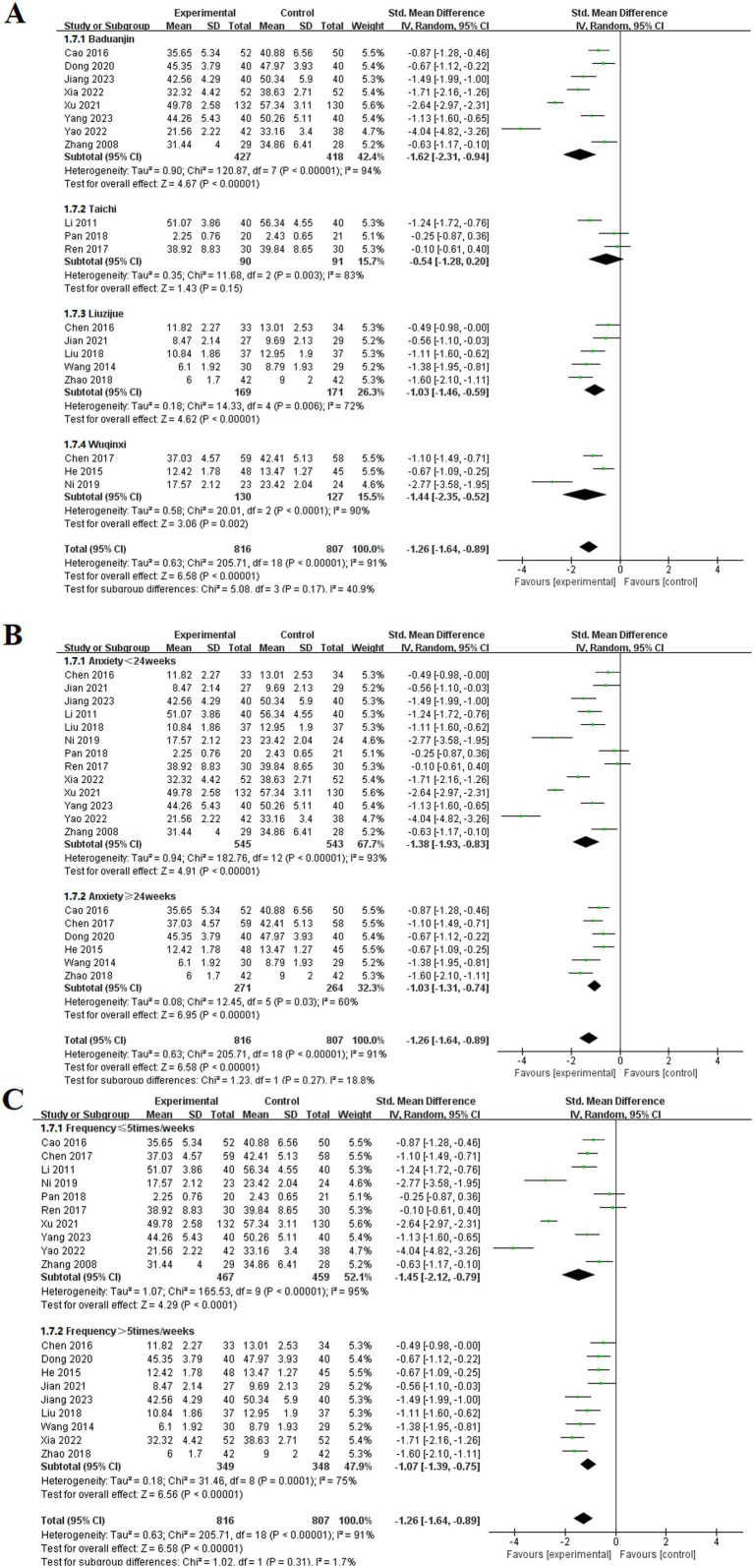
Effect of TCE on anxiety in patients with COPD. **(A)** Types of intervention; **(B)** Duration of intervention; **(C)** Frequency of intervention.

Subgroup analyses were conducted on the basis of intervention duration and frequency. In the analysis of intervention duration, the experimental group exhibited a significantly lower anxiety than did the control group before 24 weeks of TCE [MD = −1.38, 95% CI (−1.93, −0.83), *p* < 0.001]. Similarly, for interventions lasting 24 weeks or longer, the experimental group demonstrated a statistically significant alleviation of anxiety [MD = −1.03, 95% CI (−1.31, −0.74), *p* < 0.001]. The subgroup analysis based on exercise frequency indicated that both groups in the experimental cohort presented significant differences at frequencies of ≤ 5 times per week [MD = −1.45, 95% CI (−2.12, −0.79), *p* < 0.001] and > 5 times per week [MD = −1.07, 95% CI (−1.39, −0.75), *p* < 0.001] (Figure 10).

#### 3.3.8 Depression

Among the 19 included studies (9, 10, 14, 21, 22, 24–26, 36, 42, 44, 48, 53–56, 65, 66, 69), the effects of TCE on depression in patients with COPD were reported. These studies involved four types of exercises: Baduanjin, Taichi, Liuzijue, and Wuqinxi. The experimental group consisted of 816 participants, whereas the control group included 807 participants. The studies utilized SMD to compile data on anxiety symptoms using assessment tools such as the SDS, HAM-D, and HADS. The random-effects model indicated that all TCE groups showed significantly lower depression than did the control group. The results for each group included Baduanjin [MD = −1.51, 95% CI (−2.10, −0.92), *p* < 0.001], Taichi [MD = −1.11, 95% CI (−2.06, 0.17), *p* < 0.001], Liuzijue [MD = −1.12, 95% CI (−1.50, −0.74), *p* < 0.001], and Wuqinxi [MD = −0.98, 95% CI (−1.66, −0.30), *p* < 0.001].

**Figure 11:**
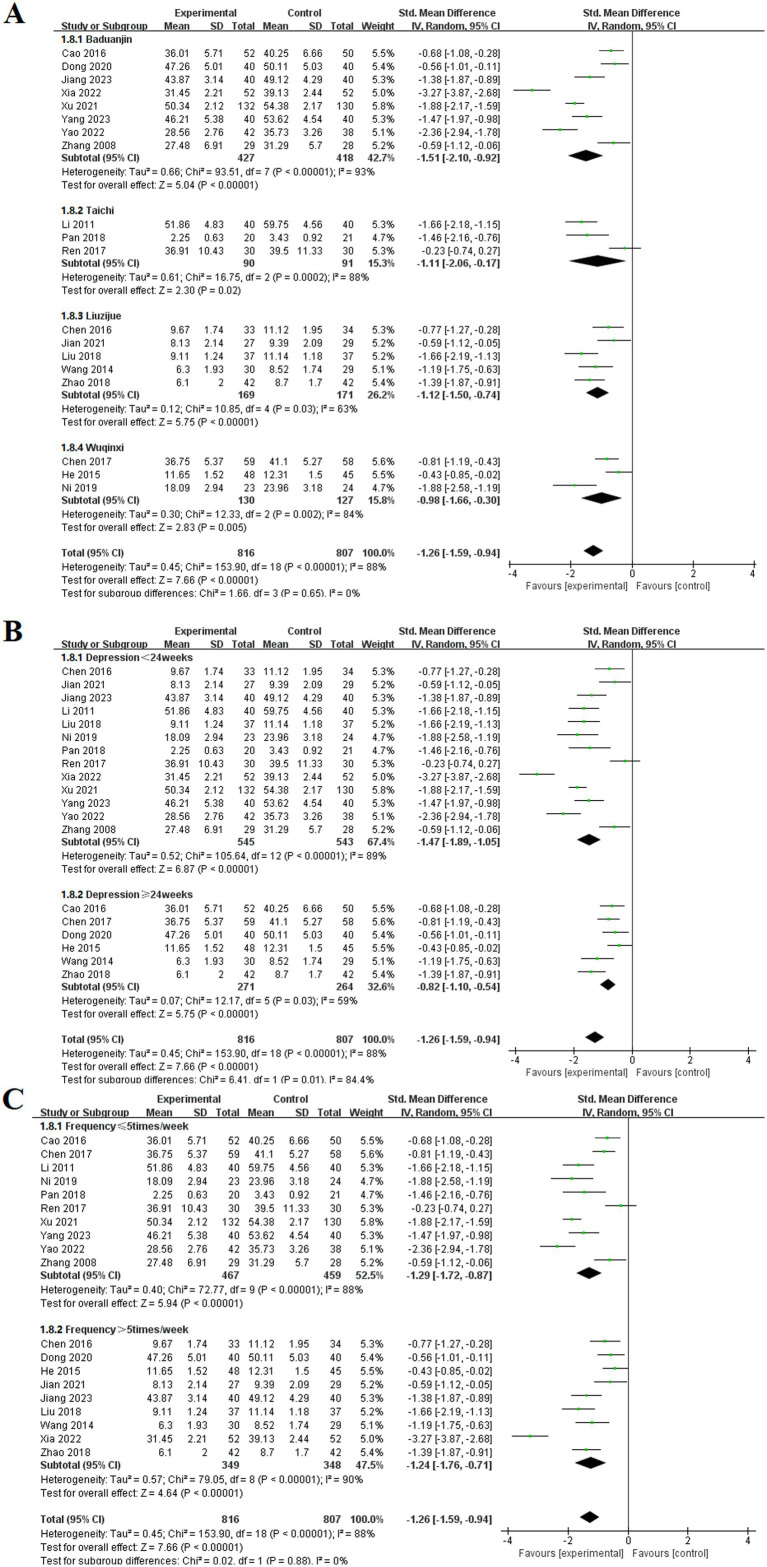
Effect of TCE on depression in patients with COPD. **(A)** Types of intervention; **(B)** Duration of intervention; **(C)** Frequency of intervention.

Subgroup analyses were conducted on the basis of intervention duration and frequency. In the analysis of intervention duration, the experimental group exhibited a significantly lower depression than the control group before 24 weeks of TCE [MD = −1.47, 95% CI (−1.89, −1.05), *p* < 0.001]. Similarly, for interventions lasting 24 weeks or longer, the experimental group demonstrated a statistically significant alleviation of depression [MD = −0.82, 95% CI (−1.10, −0.54), *p* < 0.001]. The subgroup analysis based on exercise frequency indicated that both groups in the experimental cohort presented significant differences at frequencies of ≤ 5 times per week [MD = −1.29, 95% CI (−1.72, −0.87), *p* < 0.001] and > 5 times per week [MD = −2.24, 95% CI (−1.76, −0.71), *p* < 0.001] (Figure 11).

### 3.4 Publication bias and sensitivity analysis

**Figure 12:**
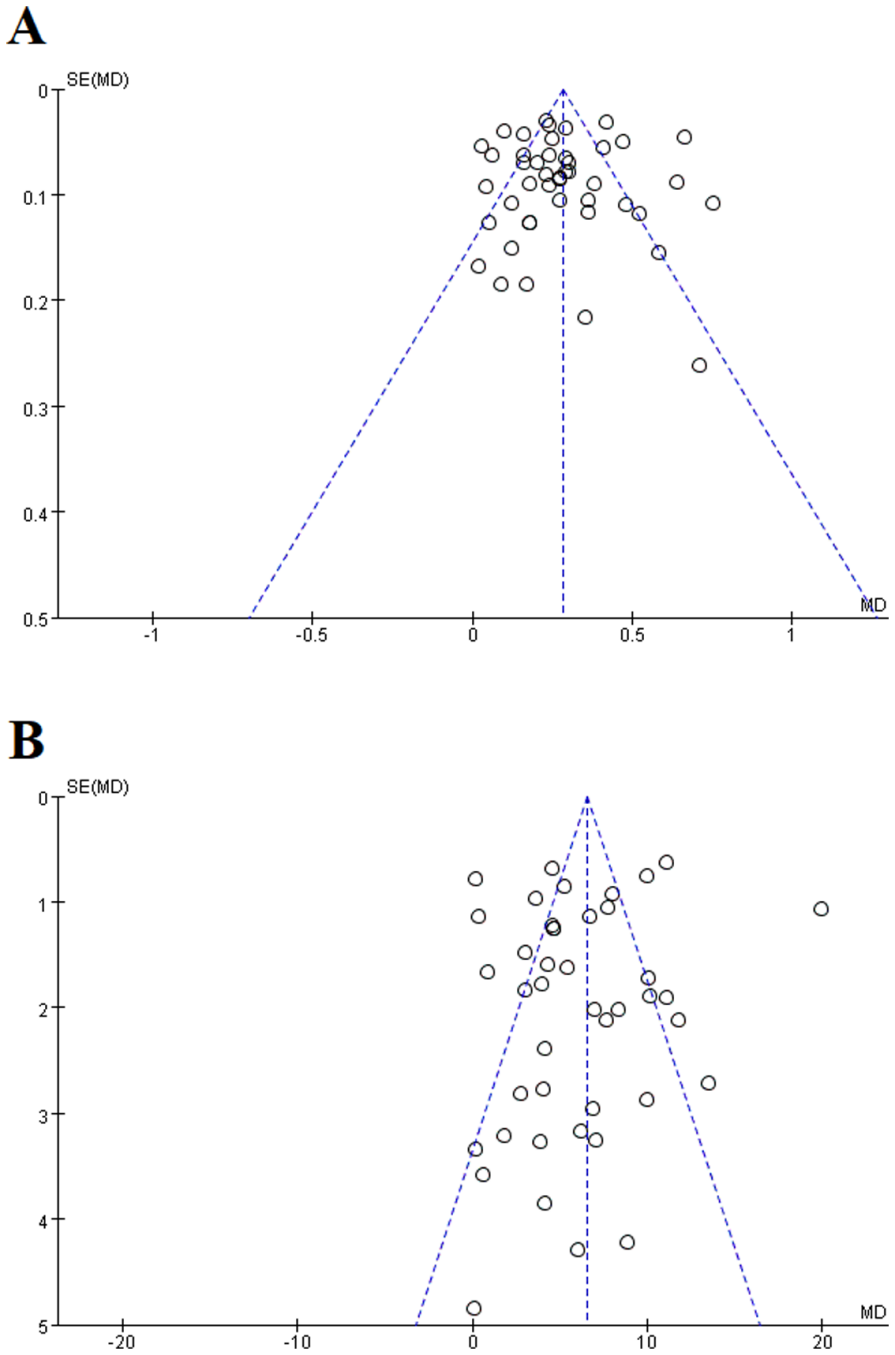
Funnel plot of FEV1, FEV1/FVC (%). **(A)** FEV1; **(B)** FEV1/FVC (%).

Given the restricted literature included, this study exclusively focused on bias in the test outcomes for FEV1 and FEV1/FVC (%). A visual inspection of the funnel plot indicated symmetry (Figure 12). To further validate these findings, the Egger’s test was conducted, yielding *p* ≥ 0.05 (*p* = 0.984), (*p* = 0.495). Consequently, no significant publication bias or other biases were observed. The results of sensitivity analysis showed that excluding individual studies did not lead to significant changes in the groups or outcomes. Consequently, the findings of the sensitivity analysis support the reliability of the study results (Supplementary Table S2).

## 4 Discussion

In this study, 67 RCTs involving 5,475 cases were included, with a focus on five exercise methods: Baduanjin, Taichi, Liuzijue, Wuqinxi, and Yijinjing. These results indicate that TCE significantly improves lung function and mental health in COPD patients. Sensitivity analysis found no substantial changes, suggesting that the included studies are relatively stable, the funnel plots and the Egger’s test indicated no publication bias in this study. However, owing to the unique characteristics of different exercise methods, heterogeneity is relatively high, which may lead to greater clinical variability. Therefore, we categorized the different exercise methods for analysis. Subgroup analysis did not significantly reduce heterogeneity, possibly because of age differences, blinding methods, and varying degrees of outcome improvement. A deeper exploration of potential sources of heterogeneity is warranted. Specifically, cultural perceptions of TCE may influence participant motivation and adherence. In regions where TCE is embedded in daily life and traditional health practices, such as in China, patients may perceive these exercises as familiar, credible, and low-barrier interventions. In contrast, in Western contexts, a lack of cultural familiarity or skepticism toward traditional practices may lead to lower adherence or different expectations. Moreover, regional disparities in healthcare infrastructure, instructor qualifications, and educational forms may further contribute to variations in intervention veracity and patient outcomes. These contextual factors, though often unreported, likely represent significant sources of heterogeneity across studies. Thus, higher-quality evidence may be needed to confirm our findings (76). The subgroup analysis clearly suggested that traditional Chinese exercises can improve various lung function parameters in COPD patients to different extents, thus alleviating disease symptoms and enhancing their quality of life.

COPD is a multifaceted systemic disease that has become a major global public health issue. It not only causes damage to lung function but also leads to peripheral muscle dysfunction, negatively impacting physical and mental health (77). Previous research has confirmed the significant health benefits of traditional Chinese exercise, indicating that regular practice can enhance aerobic capacity, strengthen respiratory muscles, improve physical fitness, regulate breathing functions, enhance gas exchange efficiency, slow the decline in lung function, and promote health-related quality of life and mental well-being. Since COPD requires long-term exercise rehabilitation interventions, the study of the effects of TCE on health outcomes has continued to expand and become a key focus for many COPD patients.

TCEs are an essential part of nonpharmacological therapies and involves a series of relaxed and controlled postures that integrate breathing control with functional movements. These exercises stimulate the body’s functional reserves following illness and promote recovery after acute conditions. They are not limited by space and do not require any special equipment or settings. With repeated practice over time, traditional Chinese exercises support maximum functional recovery, enhance neuroplasticity and proprioception, and help remodel cardiopulmonary dynamics and muscle tone. As a form of aerobic exercise, the mechanisms by which traditional Chinese exercises improve lung function in COPD patients may include reducing the spasmodic state of small arteries, preventing bronchial airway obstruction and increasing ventilation, improving lung oxygen diffusion, and increasing partial oxygen pressure. Unlike modern exercise forms such as high-intensity interval training, resistance training, or structured gym-based rehabilitation, TCEs focus on low-impact, rhythmical movements combined with breath control and mental focus. Modern exercises that emphasize physical intensity and measurable performance outcomes, often require specific facilities, equipment, or professional supervision, which can present barriers to access for older adults or those in low-resource settings. In contrast, TCEs emphasize balance, relaxation, and internal energy regulation, are low-cost, low-impact, adaptable to a wide range of physical conditions, and can be practiced independently in various environments, including at home or in community parks. Moreover, TCEs typically place greater emphasis on harmonizing the body and mind, cultivating relaxation, and promoting emotional resilience. These psychological benefits are often under-addressed in conventional exercise. For patients with COPD, who frequently experience anxiety and depression, the Physical and psychological recovery ability of TCE may offer unique advantages beyond those conferred by physical training alone. Additionally, COPD self-management education is a vital element in the implementation of traditional Chinese exercise. This educational approach incorporates disease knowledge, skill training, and strategies for behavior modification. Increasing patients’ understanding of the importance and benefits of traditional Chinese exercises can lead to improvements in quality of life, reductions in acute exacerbations and hospitalizations, changes in sedentary lifestyles, and increased motivation for exercise rehabilitation. Consequently, self-management and education play a significant role in the early identification, prevention, and rehabilitation of COPD (78). In the field of public health, traditional Chinese exercises emphasize practitioners’ cultural understanding and cognitive identification. As a practice combining exercise and meditation, they can alleviate stress, enhance emotional regulation, and improve symptoms of anxiety and depression by inhibiting the activation of the sympathetic nervous system. For example, workers often face more serious of emotional stress, and integrating traditional Chinese exercises into daily life can provide a valuable self-care method to support mental health. Similarly, incorporating traditional Chinese exercises into student groups facing academic pressure and uncertainty may help reduce stress and enhance their focus in daily life.

Traditional Chinese exercises exhibit unique compatibility, making them a versatile supplement to comprehensive treatment plans. Their simplicity and ease of learning make them particularly suitable for older adults, providing a moderate yet holistic form of exercise that encompasses breathing, cardiovascular function, endurance training, psychological regulation, and limb coordination. This enhances older adults’ ability to meet various health needs and complements medical interventions. For younger age groups, traditional Chinese exercises may offer greater benefits in regulating the autonomic nervous system. However, since the intensity of these exercises may not meet the recommended levels of aerobic activity, their effectiveness in preventing cardiovascular disease might be limited. Therefore, in the rehabilitation of COPD patients, combining traditional Chinese exercises with modern exercise regimens may be more effective than relying solely on one form of exercise. Meanwhile, the practical application of TCE in COPD management requires attention to healthcare context and cultural adaptability. In Eastern healthcare systems, where TCE is widely accepted and even reimbursed in some cases, clinicians can incorporate these interventions seamlessly into routine pulmonary rehabilitation programs. In contrast, in Western healthcare systems, successful integration may depend on exercises category, patient education, and availability of qualified instructors. Introducing culturally tailored exercise types, pilot community-based TCE programs, or integrating TCE into existing physiotherapy services could enhance feasibility and uptake. Furthermore, some researchers have evaluated improvements in patients’ physical function by analyzing results such as exercise performance, scale evaluation, neural imaging activation, and physiological indicators, which can provide more comprehensive evidence for the role of TCE in promoting COPD rehabilitation.

This review has several limitations. First, in some of the included RCTs, traditional Chinese exercises were combined with other therapies, which may have diluted the specific effects of traditional Chinese exercise. The observed effects may have been impacted by these additional therapies, and making it difficult to attribute the improvement in the disease solely to traditional Chinese exercises. Second, variations in study quality, sample size, exercise duration, frequency, intensity, outcome measures, and patient factors (e.g., participants’ proficiency, age, and compliance in performing the exercises) may have contributed to heterogeneity in the results. Most included studies did not report concealment or blinding of the allocation of participants and researchers, limiting the ability to draw clear conclusions about the effectiveness of the intervention measures. Finally, since most research has been conducted in China, the findings may not be easily generalizable to other countries. As more research in this area continues to emerge, future studies should aim to include larger, high-quality, multicenter trials. These limitations emphasize the need for future research to combine rigorously designed controlled trials, standard implementation of blinding methods, and precise statistical approaches to provide stronger evidence regarding the independent efficacy of TCE in the rehabilitation of COPD patients.

## 5 Conclusion

To summarize, this study shows that TCE have a significant influence on improving lung function, alleviating anxiety and depression in COPD patients. Compared with drug intervention, TCEs are simple, inexpensive, easy to learn and practice, and not limited by the venue. They are one of the important means to alleviate the social and economic burden caused by COPD, providing more choices for clinical treatment. This study not only highlights the nonpharmacological intervention value of TCE but also provides new directions for psychological intervention, which deserves wider promotion.

## Data Availability

The original contributions presented in the study are included in the article/Supplementary material, further inquiries can be directed to the corresponding author.
