# Supplementary material for: Effects of traditional Chinese exercise on lung function and mental health in patients with COPD: a systematic review and meta-analysis
Source: Front Public Health. 2025 Jul 10;13:1612741. doi: 10.3389/fpubh.2025.1612741 (PMC12286970; doi:10.3389/fpubh.2025.1612741)
Supplement: Supplementary file 1 [file Data_Sheet_1.pdf]

Author (year)	TG			CG			Intervention time	Outcome indicators
	Sample size	Age (M ± SD)	Treatment measures	Sample size	Age (years)	Treatment measures		
Cao 2016 [9]	52	70.83±5.96	Baduanjin 4×30 min/week	50	70.14±5.71	Usual care + Daily activities	24 weeks	FEV_1, $FEV_{1\%}$, FEV1/FVC, SAS, SDS
Dong 2020 [10]	40	65.2±6.0	Baduanjin 1x40 min/day + Comprehensive Care	40	65.0±5.7	Usual care	24 weeks	CAT, SAS, SDS
Hou 2017 [11]	25	63.34±5.95	Baduanjin 1x30 min/day + Pulmonary Rehabilitation	23	63.77±6.1	Usual care + Pulmonary Rehabilitation	12 weeks	FEV_1, FVC, FEV1/FVC, 6MWT
Huang 2016 [12]	31	67.78±3.46	Baduanjin 1x30 min/day	30	65.31±4.56	Usual care	12 weeks	FEV_1, $FEV_{1\%}$, FVC, FEV1/FVC
Huang 2021 [13]	38	51.30±3.24	Baduanjin 1x30 min/day + Pulmonary Rehabilitation	38	51.46±3.28	Usual care + Pulmonary Rehabilitation	12 weeks	FEV_1, 6MWT, CAT
Jiang 2023 [14]	40	57.28±6.43	Baduanjin 1x25 min/day	40	57.45±6.39	Usual care + Health education	12 weeks	FEV_1, FVC, FEV1/FVC, 6MWT, CAT, SAS, SDS
Liu 2013 [15]	40	59.77±7.08	Baduanjin 1x30 min/day	40	60.67±6.95	Usual care	12 weeks	$FEV_{1\%}$, FEV1/FVC, 6MWT
LiuY 2021 [16]	37	58.59±1.89	Baduanjin 1x40 min/day + Acupressure therapy	37	58.71±1.92	Usual care + Acupressure therapy	24 weeks	FEV_1, FVC, CAT
Ma 2020 [17]	30	65.5±5.0	Baduanjin 4x30 min/week	30	65.8±5.1	Usual care	18 weeks	$FEV_{1\%}$, FVC, FEV1/FVC, 6MWT
Ma 2022 [18]	41	69.83±3.51	Baduanjin 1x30 min/day	41	69.35±2.84	Usual care + Daily activities	12 weeks	FEV_1, FVC, 6MWT
Wang 2018 [19]	38	63.17±9.95	Baduanjin 1x30 min/day	38	63.67±9.75	Usual care	24 weeks	FEV_1, $FEV_{1\%}$, FVC, FEV1/FVC, 6MWT
Wang 2022 [20]	40	59.76±7.59	Baduanjin 2x30 min/day + Pulmonary Rehabilitation	40	58.95±7.18	Usual care + Pulmonary Rehabilitation	12 weeks	FEV_1, FVC, 6MWT
Xu 2021 [21]	132	68.89±9.67	Baduanjin 5x30 min/week	130	69.01±9.72	Usual care + Health education	12 weeks	FEV_1, FVC, FEV1/FVC, 6MWT, SAS, SDS
Xia 2022 [22]	52	67.42±0.44	Baduanjin 1x40 min/day	52	68.29±9.38	Routine care + Health education	16 weeks	FEV_1, FVC, SAS, SDS
Yu 2019 [23]	41	62.3±1.2	Baduanjin 1x30 min/day	41	62.3±1.5	Usual care	12 weeks	FEV_1, $FEV_{1\%}$, FVC, FEV1/FVC, 6MWT
Yao 2022 [24]	42	64.55±3.47	Baduanjin 3x15 min/week + Comprehensive	38	66.47±4.19	Usual care	8 weeks	6MWT, CAT, SAS, SDS

Author (year)	TG			CG			Intervention time	Outcome indicators
	Sample size	Age (M ± SD)	Treatment measures	Sample size	Age (years)	Treatment measures		
			Care					
Yang 2023 [25]	40	47.88±2.49	Baduanjin 4×40 min/week	40	48.75±2.41	Usual care + Daily activities	4 weeks	FEV_1, $FEV_{1\%}$, FVC, 6MWT, CAT, SAS, SDS
Zhang 2008 [26]	29	69.90±4.73	Baduanjin 3x30 min/week	28	69.68±8.66	Usual care	8 weeks	SAS, SDS
Zhang 2019 [27]	30	65.46±6.74	Baduanjin 1x30 min/day	30	64.46±6.74	Usual care	24 weeks	$FEV_{1\%}$, FEV1/FVC, 6MWT
Zhu 2014 [28]	63	69.0±8.7	Baduanjin 2x30 min/day	60	68.0±9.2	Usual care	24 weeks	FEV_1, $FEV_{1\%}$, FVC, FEV1/FVC, 6MWT
Chen 2020 [29]	63	51.9±2.9	Taichi 1x60 min/week	63	51.3±2.6	Usual care + Daily activities	8 weeks	$FEV_{1\%}$, 6MWT, CAT
Chi 2021 [30]	108	68.03±6.58	Taichi 1x30 min/day	108	67.43±7.34	Usual care	52 weeks	FEV_1, FVC, FEV1/FVC
Deng 2016 [31]	63	66.6±8.8	Taichi 1x30 min/day	63	67.1±9.0	Usual care	24 weeks	$FEV_{1\%}$, 6MWT
Du 2013 [32]	36	65.23±8.37	Taichi 2x30 min/day	38	64.48±6.54	Usual care	12 weeks	$FEV_{1\%}$, FEV1/FVC, 6MWT, CAT
Gu 2012 [33]	33	67±8	Taichi 3x60 min/week	30	69±9	Usual care	12 weeks	FEV_1, $FEV_{1\%}$, FVC, FEV1/FVC, 6MWT
He 2020 [34]	45	NA	Taichi 5x45 min/week	45	NA	Usual care + Daily activities	4 weeks	FEV_1, FVC, FEV1/FVC, 6MWT
Hu 2020 [35]	42	62.09±3.80	Taichi 2x35 min/day	42	62.20±3.51	Usual care	12 weeks	FEV_1, $FEV_{1\%}$, FEV1/FVC
Li 2011 [36]	40	64.56±4.73	Taichi 3x30 min/week	40	62.68±5.76	Usual care	12 weeks	SAS, SDS
Li 2012 [37]	30	72.0±2.5	Taichi 1x60 min/day + Pulmonary Rehabilitation Training	30	73.6±3.0	Usual care + Pulmonary Rehabilitation Training	24 weeks	FEV_1, FVC, FEV1/FVC
Li 2016 [38]	20	60.3±6.9	Taichi 3x40 min/week	20	60.3±6.9	Usual care	12 weeks	FEV_1, $FEV_{1\%}$, FEV1/FVC, 6MWT
Li 2019 [39]	26	66±2.8	Taichi 5x30 min/week + Pulmonary Rehabilitation Training	23	65±2.6	Usual care + Pulmonary Rehabilitation Training	12 weeks	FEV_1, FVC, FEV1/FVC
Liu 2021 [40]	41	54.92±4.06	Taichi 1x30 min/day	40	54.69±3.81	Usual care	24 weeks	FEV_1, FVC, FEV1/FVC, 6MWT
Liu 2019 [41]	50	53.5±6.1	Taichi 2x30 min/day	50	53.1±5.6	Usual care	54 weeks	$FEV_{1\%}$, FEV1/FVC, 6MWT, CAT

**Author (year)**	**TG**			**CG**			**Intervention time**	**Outcome indicators**
	Sample size	Age (M ± SD)	Treatment measures	Sample size	Age (years)	Treatment measures		
Pan 2018 [42]	20	NA	Taichi 3x30 min/week	21	NA	Usual care + Health education	8 weeks	FEV1, $FEV_{1\%}$, FVC, 6MWT, CAT, HADS
Peng 2020 [43]	40	NA	Taichi 1x30 min/day	40	NA	Usual care	24 weeks	FEV_1, FVC, FEV1/FVC, 6MWT, CAT
Ren 2017 [44]	30	59.2±4.5	Taichi 2x60 min/week	30	58.9±4.2	Usual care + Health education	12 weeks	FEV_1, FVC, FEV1/FVC, 6MWT, CAT SAS, SDS
Zhang 2014 [45]	18	68.02±6.91	Taichi 2x60 min/day	18	66.71±5.84	Usual care	52 weeks	FEV_1, $FEV_{1\%}$, FEV1/FVC, 6MWT, CAT
ZhangY 2019 [46]	29	49.24±2.19	Taichi 1x60 min/day + Pulmonary Rehabilitation Training	29	50.13±2.08	Usual care + Pulmonary Rehabilitation Training	108 weeks	FEV1, $FEV_{1\%}$, FVC
Chen 2008 [47]	21	71.76±7.31	Liuzijue 1x30 min/day	19	73.32±6.33	Usual care	12 weeks	FEV_1, $FEV_{1\%}$, FEV1/FVC
Chen 2016 [48]	33	68.75±8.67	Liuzijue 2x30 min/day	34	69.31±7.54	Usual care	8 weeks	$FEV_{1\%}$, FVC, FEV1/FVC, CAT, HADS
Cao 2022 [49]	29	69.78±2.68	Liuzijue 1x20 min/day	31	69.30±2.83	Usual care	24 weeks	FEV_1, $FEV_{1\%}$, FEV1/FVC
Deng 2020 [50]	30	76.53±8.59	Liuzijue 2x15 min/day	32	76.59±7.69	Usual care	12 weeks	FEV_1, $FEV_{1\%}$, FEV1/FVC
HouMY 2017 [51]	50	NA	Liuzijue 2x30 min/day	49	NA	Usual care	24 weeks	FEV_1, FEV1/FVC
Ji 2019 [52]	28	63.75±5.48	Liuzijue 1x30 min/day	29	64.52±5.68	Usual care	12 weeks	$FEV_{1\%}$, FVC, FEV1/FVC
Jian 2021 [53]	30	59.44±6.13	Liuzijue 2x30 min/day	30	62.02±6.24	Usual care	12 weeks	$FEV_{1\%}$, HADS
Wang 2014 [54]	30	70.1±11.03	Liuzijue 1x30 min/day	30	71.1±8.4	Usual care	52 weeks	HAMA, HAMD
Zhao 2018 [55]	42	67.7±10.8	Liuzijue 2x30 min/day + Respiratory rehabilitation	42	68.2±11.8	Usual care + Respiratory rehabilitation	52 weeks	FEV_1, FEV1/FVC, HAMA, HAMD
Liu 2018 [56]	38	57.54±5.14	Liuzijue 2x30 min/day + Acupressure therapy	37	68.11±5.58	Usual care	12 weeks	FEV1/FVC, CAT, HAMA, HAMD
Ju 2022 [57]	80	71.04±8.22	Liuzijue 2x30 min/day + Acupressure therapy	80	70.45±9.37	Usual care + Health education	54 weeks	FEV_1, FVC, FEV1/FVC, CAT, 6MWT
Li 2024 [58]	40	65.33±2.41	Liuzijue 2x30 min/day + acupressure therapy	40	65.29±2.37	Usual care	12 weeks	FEV_1, FVC
Sun 2019 [59]	56	5.45±6.24	Liuzijue 2x10 min/day	56	4.78±6.12	Usual care	24 weeks	FEV_1, $FEV_{1\%}$, 6MWT

Author (year)	TG			CG			Intervention time	Outcome indicators
	Sample size	Age (M ± SD)	Treatment measures	Sample size	Age (years)	Treatment measures		
Wang 2023 [60]	20	66.20±7.82	Liuzijue 5x30 min/week + Inspiratory muscle training	20	67.13±8.62	Usual care + Inspiratory muscle training	8 weeks	$FEV_{1\%}$, CAT, 6MWT
XuL 2021 [61]	18	NA	Liuzijue 2x30 min/week	20	NA	Usual care	24 weeks	FEV_1, $FEV_{1\%}$, FEV1/FVC, CAT, 6MWT
Yan 2023 [62]	30	76.62±2.69	Liuzijue 5x30 min/week	30	75.3±2.67	Usual care	12 weeks	FEV_1, FEV1/FVC, 6MWT
Zhao 2012 [63]	23	75±7	Liuzijue 2x20 min/day	22	75±7	Usual care	24 weeks	FEV_1, $FEV_{1\%}$, 6MWT
ZhangFR 2019 [64]	62	71.3±2.96	Liuzijue 1x30 min/day	58	72.9±3.25	Usual care	12 weeks	FEV1, 6MWT
Chen 2017 [65]	60	52.14±6.38	Wuqinxi 5x30 min/week	60	53.27±7.12	Usual care	24 weeks	6MWT, SAS, SDS
He 2015 [66]	48	58.66±7.56	Wuqinxi 2x30 min/day	45	58.64±7.52	Usual care	24 weeks	FEV1/FVC, CAT, HAMA, HAMD
Gao 2017 [67]	36	67.14±9.08	Wuqinxi 1x30 min/day	35	66.03±8.18	Usual care + Daily activities	12 weeks	FEV_1, $FEV_{1\%}$, FEV1/FVC, 6MWT
Liu 2020 [68]	50	74.24±9.1	Wuqinxi 1x45 min/day	50	67.72±9.26	Usual care	12 weeks	FEV_1, $FEV_{1\%}$, FVC, FEV1/FVC, 6MWT
Ni 2019 [69]	23	51.78±4.02	Wuqinxi 5x60 min/week	24	51.08±4.49	Usual care	12 weeks	HAMA, HAMD
Sun 2021 [70]	40	71.29±11.12	Wuqinxi 1x30 min/day + music therapy of five elements	40	71.76±11.11	Usual care	8 weeks	FEV_1, CAT, FEV1/FVC
Wei 2015 [71]	48	58.66±7.56	Wuqinxi 1x30 min/day	45	58.64±7.52	Usual care	24 weeks	$FEV_{1\%}$, FEV1/FVC
Xiao 2023 [72]	58	59.89±3.42	Wuqinxi 1x30 min/day	58	60.37±2.86	Usual care + Health education	12 weeks	FEV_1, FVC, FEV1/FVC, 6MWT
Zang 2017 [73]	32	54.1±11.3	Wuqinxi 1x20 min/day+ Simple breath exercises	36	58.7±10.4	Usual care	24 weeks	$FEV_{1\%}$, 6MWT, CAT
Gao 2016 [74]	55	71.42±10.45	Yijinjing 2x60 min/day	57	74.24±10.34	Usual care	24 weeks	FEV_1, $FEV_{1\%}$, FEV1/FVC, 6MWT
Zhang 2016 [75]	20	61.77±4.07	Yijinjing 2x60 min/day	25	59.35±5.27	Usual care	24 weeks	FEV_1, $FEV_{1\%}$, FEV1/FVC, 6MWT
