# Supplementary material for: Effects of traditional Chinese exercise on lung function and mental health in patients with COPD: a systematic review and meta-analysis
Source: Front Public Health. 2025 Jul 10;13:1612741. doi: 10.3389/fpubh.2025.1612741 (PMC12286970; doi:10.3389/fpubh.2025.1612741)
Supplement: Supplementary file 2 [file Data_Sheet_2.pdf]

A

```
. metabias _ES _seES,egger graph

Note: data input format theta se_theta assumed

Egger's test for small-study effects:
Regress standard normal deviate of intervention
  effect estimate against its standard error

.
Number of studies =  45                         Root MSE      =   2.432
------------------------------------------------------------------------------
     Std_Eff | Coefficient  Std. err.      t    P>|t|     [95% conf. interval]
-------------+----------------------------------------------------------------
       slope |   .2793437   .0504072    5.54   0.000     .1776879    .3809995
        bias |   .0152789   .7788991    0.02   0.984    -1.555521    1.586079
------------------------------------------------------------------------------

Test of H0: no small-study effects          P = 0.984

.
```

B

```
. metabias _ES _seES,egger graph

Note: data input format theta se_theta assumed

Egger's test for small-study effects:
Regress standard normal deviate of intervention
  effect estimate against its standard error

.
Number of studies =  43                         Root MSE      =   3.201
------------------------------------------------------------------------------
     Std_Eff | Coefficient  Std. err.      t    P>|t|     [95% conf. interval]
-------------+----------------------------------------------------------------
       slope |   7.398396   1.386002    5.34   0.000     4.599307    10.19748
        bias |  -.6891117   .9998669   -0.69   0.495    -2.708384     1.33016
------------------------------------------------------------------------------

Test of H0: no small-study effects          P = 0.495

.
```

Supplementary Appendix Figure 1: Eggers test results of FEV1 and FEV1/FVC (%) outcome indicators. (A) FEV1 ;(B) FEV1/FVC (%).

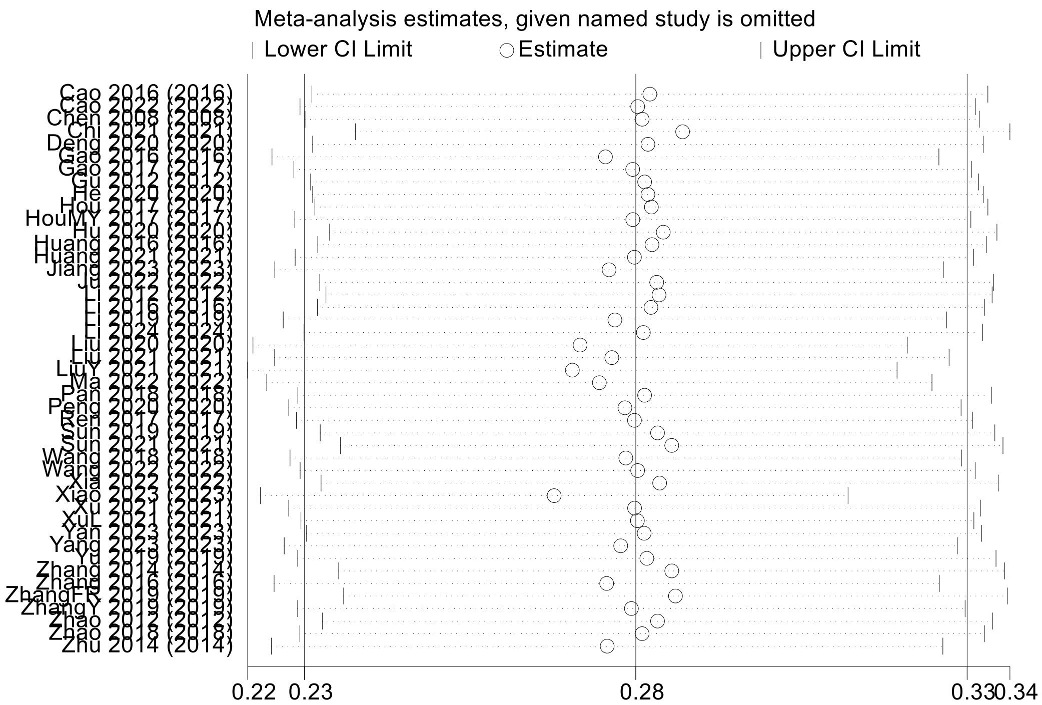

Supplementary Appendix Figure 2: Sensitivity analysis of FEV1 outcome indicators included in the study

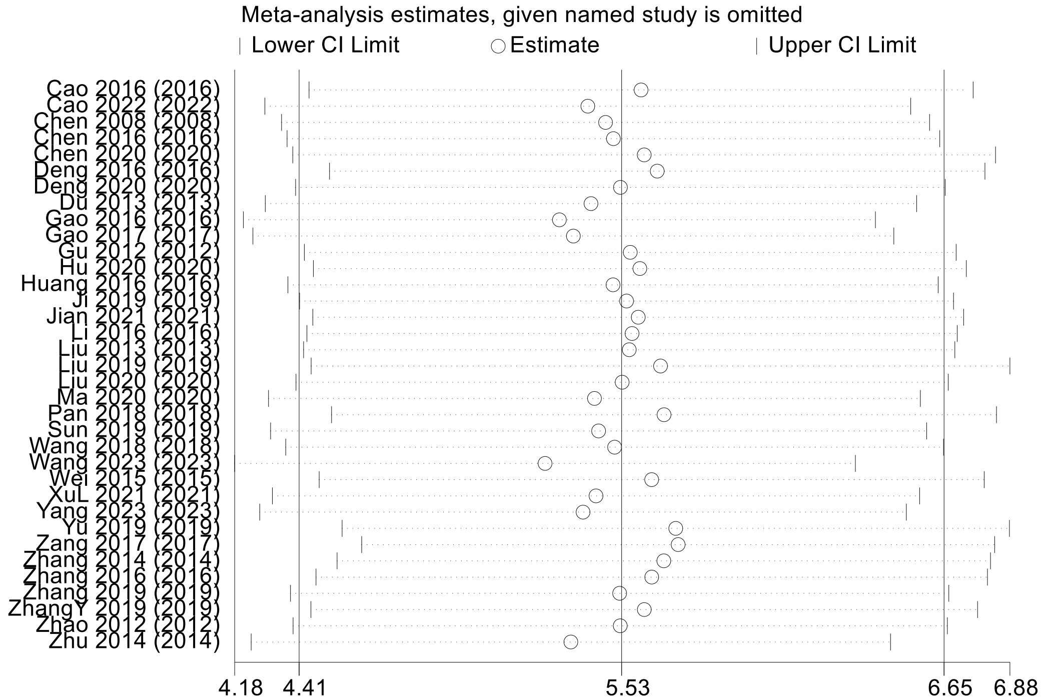

Supplementary Appendix Figure 3: Sensitivity Analysis of FEV1% Outcome Indicators for Inclusion in the Study

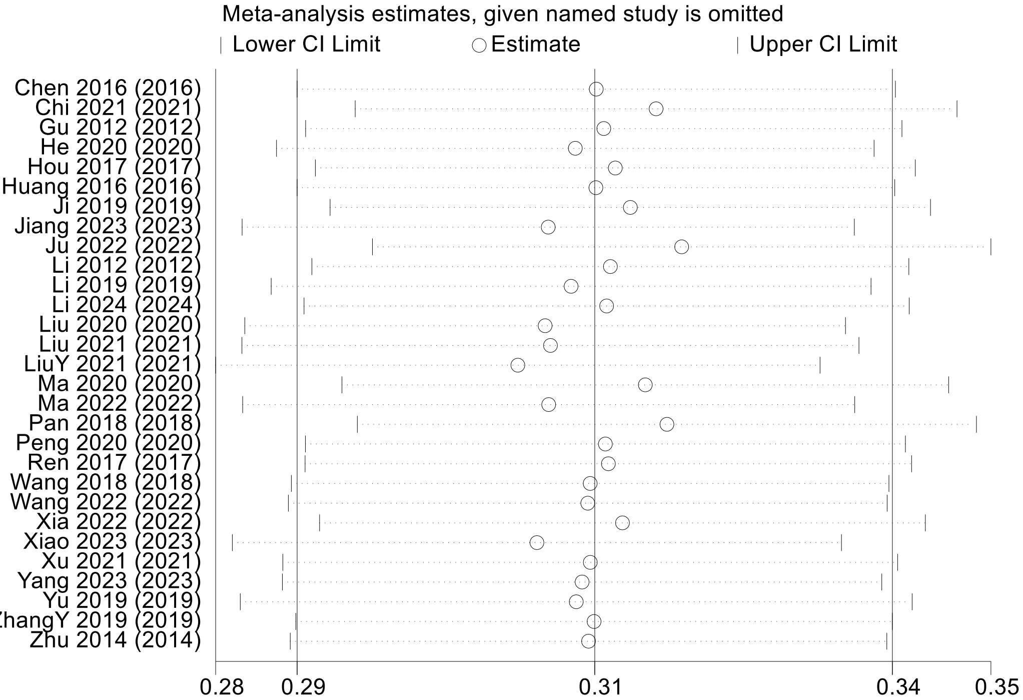

Supplementary Appendix Figure 4: Sensitivity Analysis of FVC Outcome Indicators for Inclusion in the Study

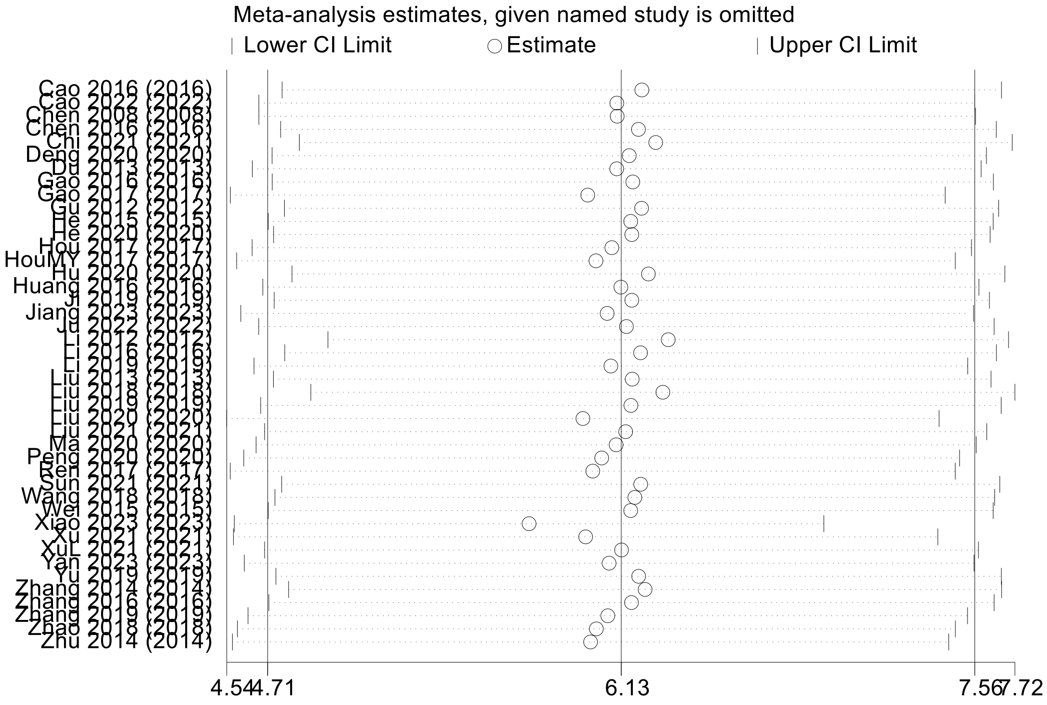

Supplementary Appendix Figure 5: Sensitivity Analysis of FEV1/FVC (%) Outcome Indicators for Inclusion in the Study

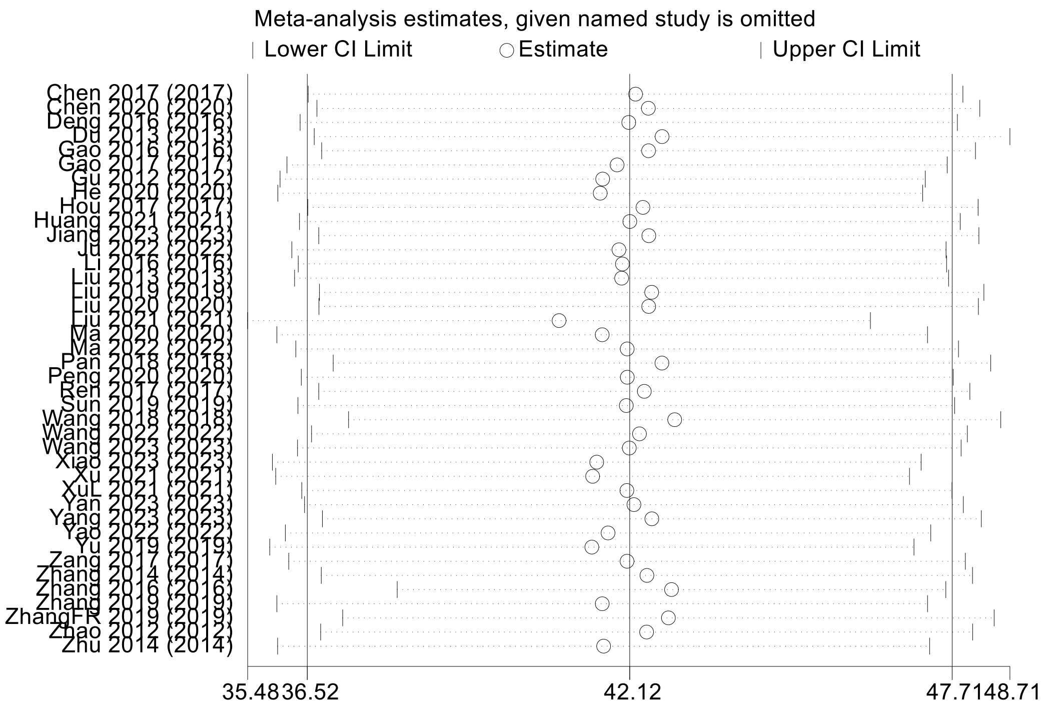

Supplementary Appendix Figure 6: Sensitivity Analysis of 6MWT Outcome Indicators for Inclusion in the Study

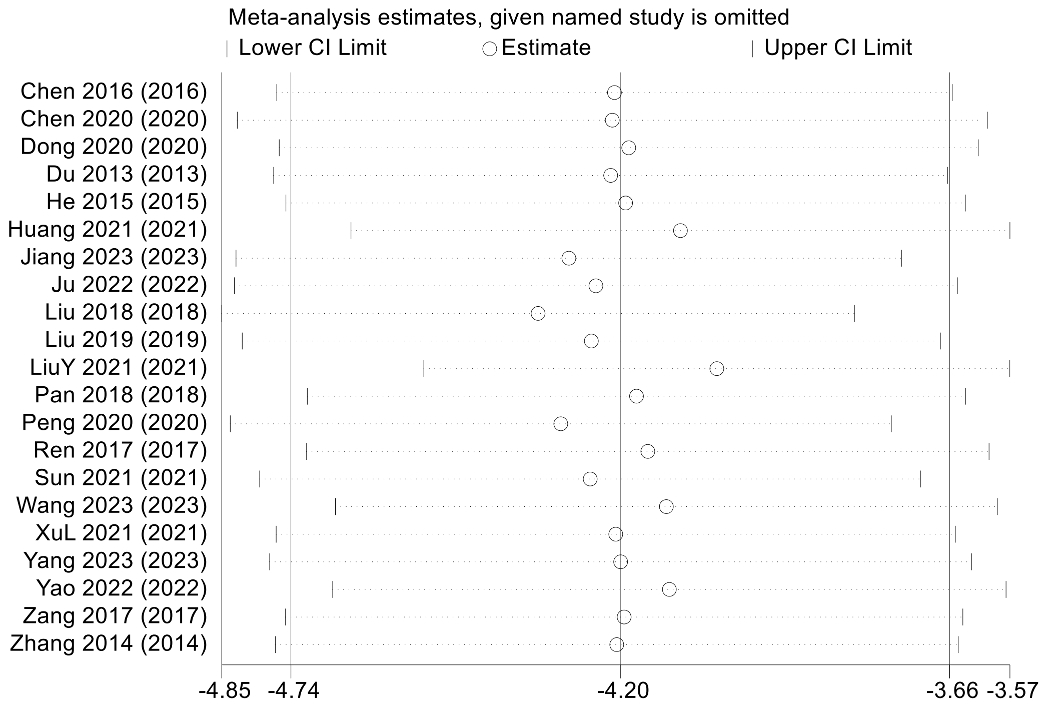

Supplementary Appendix Figure 7: Sensitivity Analysis of CAT Outcome Indicators for Inclusion in the Study

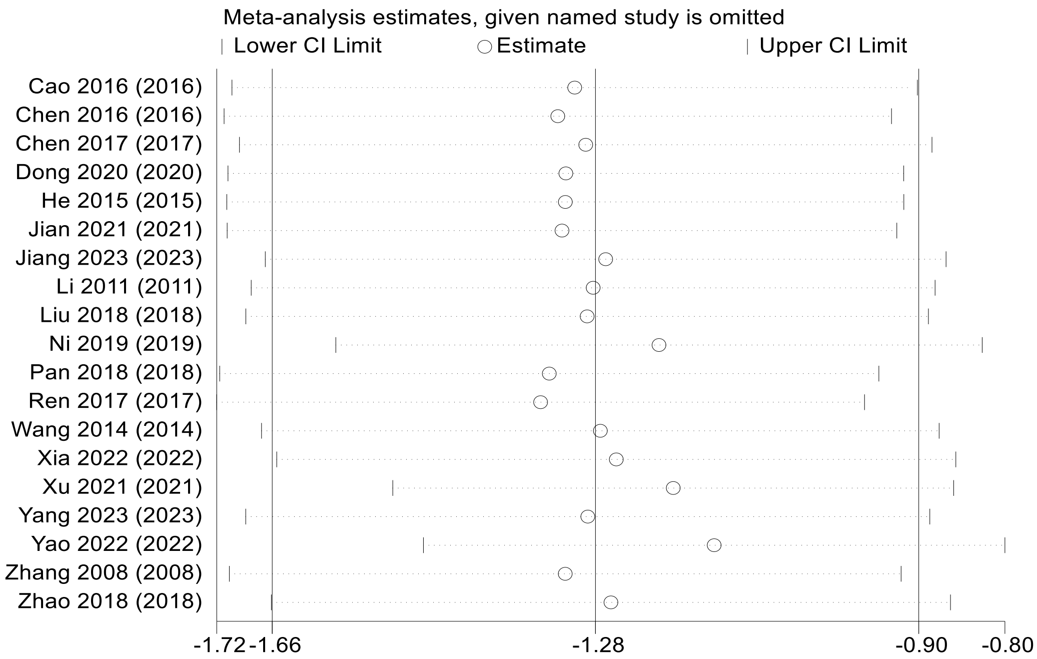

Supplementary Appendix Figure 8: Sensitivity Analysis of anxiety Outcome Indicators for Inclusion in the Study

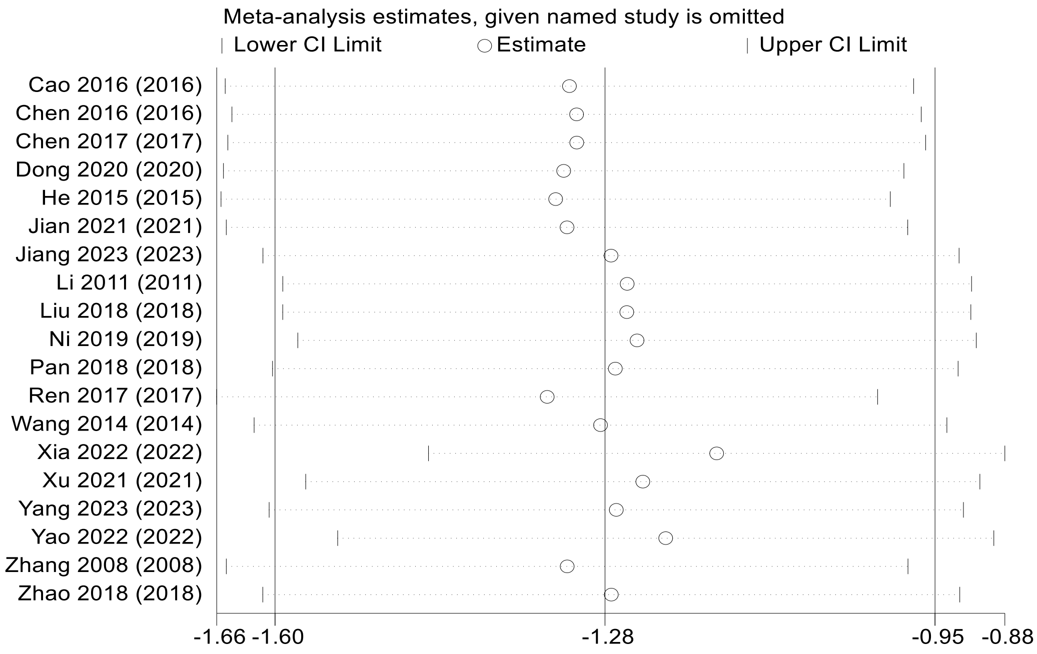

Supplementary Appendix Figure 9: Sensitivity Analysis of depression Outcome Indicators for Inclusion in the Study
